# Generalized Geographically Weighted Regression Model within a Modularized Bayesian Framework*

**DOI:** 10.1214/22-BA1357

**Published:** 2023-06-01

**Authors:** Yang Liu, Robert J. B. Goudie

**Affiliations:** † MRC Biostatistics Unit, University of Cambridge, UK; ‡ MRC Biostatistics Unit, University of Cambridge, UK

**Keywords:** geographically weighted regression, modularized Bayesian, cutting feedback, model misspecification, power likelihood, Primary 62F15, 62J12

## Abstract

Geographically weighted regression (GWR) models handle geographical dependence through a spatially varying coefficient model and have been widely used in applied science, but its general Bayesian extension is unclear because it involves a weighted log-likelihood which does not imply a probability distribution on data. We present a Bayesian GWR model and show that its essence is dealing with partial misspecification of the model. Current modularized Bayesian inference models accommodate partial misspecification from a single component of the model. We extend these models to handle partial misspecification in more than one component of the model, as required for our Bayesian GWR model. Information from the various spatial locations is manipulated via a geographically weighted kernel and the optimal manipulation is chosen according to a Kullback–Leibler (KL) divergence. We justify the model via an information risk minimization approach and show the consistency of the proposed estimator in terms of a geographically weighted KL divergence.

## 1 Introduction

Conventional regression models have been widely used in various studies to infer the association between variables. While basic regression models often assume an independent sampling scheme, geographical dependence must be taken into consideration when the dataset or sampling scheme has a spatial structure. Therefore,rather than assuming a constant association between variables with constant coefficients, models with geographically-variable coefficients have been proposed for this purpose. Suppose we have observations (*X_i_*, *Y_i_*) at sampling location *i* with coordinates (*u_i_*, *v_i_*), *i* = 0,…, *n*. We assume that the unknown true data generating process of the outcome *Y_i_*, given the covariate vector *X_i_*, is ${\overset{ˇ}{p}}_{i}\left(Y_{i}|X_{i}\right)$ at a particular location *i*. To model ${\overset{ˇ}{p}}_{i}$, we assume a generalized linear model (GLM) 𝔼(*Y*_*i*_|*X_i_*) = *g*^−1^ (*X*_*i*_*φ*(*u_i_*, *v_i_*)), with link function *g*, and where the coefficient *φ*(*u*, *v*) is a smooth function with respect to (*u*, *v*). For simplicity, we define *φ_i_* ≡ *φ*(*u_i_*, *v_i_*) for location *i*.

In addition to the coefficient *φ_i_*, for some generalized linear regression models, such as negative binomial or beta regression, for each location *i* there is an additional parameter *θ_i_* that determines the variability (scale) of the distribution. The additional parameter *θ_i_* is usually regarded as a nuisance parameter. This variability could be attributed to sampling or measurement errors, which may be different at different locations. We assume throughout this paper that *θ_i_* is similar, but not the same, across spatial locations but the variability is not spatially smooth. For instance, consider a variability induced by a difference in measurement equipment: each location may have arbitrarily used different measurement equipment, and consequently the variabilities of observations at different locations are not constant but also not spatially smooth. We denote the likelihood of the GLM as *p*(*Y_i_*|*θ_i_*, *φ_i_*) at location *i*. For example, in the case of a negative binomial likelihood, with a log link function, the likelihood is:

(1.1) $$p\left(Y_{i}|\theta_{i},\varphi_{i}\right)=\frac{\Gamma \left(Y_{i}+\theta_{i}^{-1}\right)}{\Gamma \left(\theta_{i}^{-1}\right)\Gamma \left(Y_{i}+1\right)}{\left(\frac{1}{1+\theta_{i}\text{exp}\left(X_{i}\varphi_{i}\right)}\right)}^{\theta_{i}^{-1}}{\left(\frac{\theta_{i}\text{exp}\left(X_{i}\varphi_{i}\right)}{1+\theta_{i}\text{exp}\left(X_{i}\varphi_{i}\right)}\right)}^{Y_{i}}.$$

We assume that a single location *i* = 0 is of primary interest, and our first aim is to estimate *φ*_0_ and *θ*_0_ at this location. We will then consider the case when multiple locations are of interest.

Several modelling approaches have been proposed for geographically variable coefficients. One class of approaches involves clustering locations into groups and considering a group-wise estimation of the coefficients. For example, Li and Sang (2019) proposed spatially clustered coefficient (SCC) regression that adds a penalty term to the residual sum of squares such that differences of coefficients for neighbouring locations are penalized and consequently locations may share the same coefficient. Sugasawa and Murakami (2021) proposed a partially clustered regression that allocates locations into groups, with locations sharing the same coefficients within a group. Luo et al. (2021) proposed Bayesian spatially clustered coefficient regression, with the spatial clustering defined via the connected components of an undirected graph induced by a spanning tree. This class of models has also been extended to spatial vector autoregressive regression (Yan et al., 2021). Another class of approaches are the Bayesian spatially varying coefficient (SVC) model (Gelfand et al., 2003) and its extensions (e.g., Paez et al., 2005; Finley et al., 2007; Berrocal et al., 2010; Reich et al., 2010; Zhao et al., 2020; Dambon et al., 2021). These have been developed within a standard Bayesian framework with geographically varying associations 𝔼(*Y_i_*|*X_i_*) = *X*_*i*_*φ_i_*. SVC models induce geographical dependence via a random spatial adjustment to coefficients, such as *φ_i_* = *φ_fix_*+*φ*_*random*,*i*_, where ${\left\{\varphi_{random,i}\right\}}_{i=1}^{n}$ are modeled by a Gaussian random field with a covariance structure corresponding to the geographical dependence of *n* locations. SVC models share the power of hierarchical modeling (Gelfand and Banerjee, 2017) via their similarity to spatial hierarchical models, which use a random Gaussian field to model the regression error (e.g., Zhu et al., 2005; Lin, 2010; Afroughi et al., 2011; Fuglstad et al., 2015; Utazi et al., 2019; Marques et al., 2020). SVC models do not involve any geographical weight function so the probability density is always proper and standard Bayesian inference can be applied. However, the sampling of the posterior distribution under SVC models can be challenging because the dimension of parameters (i.e., ${\left\{\varphi_{random,i}\right\}}_{i=1}^{n}$) increases with the number of locations, making both sampling parameters and inverting the spatial covariance matrix computationally difficult. This issue can be avoided in the Gaussian case because ${\left\{\varphi_{random,i}\right\}}_{i=1}^{n}$ can be integrated out to obtain the marginal likelihood of *φ_fix_* explicitly and the conditional posterior of ${\left\{\varphi_{random,i}\right\}}_{i=1}^{n}$ given *φ_fix_* is analytically tractable. However, in generalized linear models marginalization of ${\left\{\varphi_{random,i}\right\}}_{i=1}^{n}$ is not usually feasible, e.g. for binary and Poisson (Banerjee et al., 2008), and so sampling and computation could be problematic in practice due to the high dimension of the parameters if we have lots of locations (Sugasawa and Murakami, 2021). To address this computational issue, an efficient approach named PICAR was recently developed (Lee and Haran, 2022). The idea is to discretize the underlying spatial random field on a triangular mesh to reduce the dimension.

Other attractive and simpler alternatives are geographically weighted regression (GWR) models (e.g., Fotheringham et al., 1996; Brunsdon et al., 1996) and its extensions (e.g., Nakaya et al., 2005; Chen et al., 2012; da Silva and Rodrigues, 2014; da Silva and de Oliveira Lima, 2017; Mu et al., 2018; Liu et al., 2018; Li and Fotheringham, 2020; Tasyurek and Celik, 2020; Wu et al., 2021), which have been widely adopted in many spatial application areas (e.g., Windle et al., 2009; Duan and Li, 2016; Mayfield et al., 2018; Wang et al., 2019; Wu, 2020; Mohammed et al., 2022). GWR models use the first law of geography to justify additionally using data that are sampled from neighbouring locations when we have insufficient samples at a location of interest *i* = 0 to accurately estimate parameters at this location using only data from this location. The first law of geography states that ‘everything is related to everything else, but near things are more related than distant things’ (Tobler, 1970). “Borrowing” samples from neighbouring locations to support the estimation of *φ*_0_ should decrease the variance of estimates, although bias might be introduced.

For now, assume we have *m* observations *Y*_*i*,1:*m*_ = (*Y*_*i*,1_, *Y*_*i*, 2_,…, *Y*_*i*,*m*_) at each location *i*, with *i* = 0,…, *n*. The complete set of observations is *Y*_0:*n*,1:*m*_ = (*Y*_0,1:*m*_, *Y*_1,1:*m*_,…, *Y*_*n*,1:*m*_) with corresponding location-specific parameters *θ*_0:*n*_ = (*θ*_0_,…, *θ_n_*). We assume that *Y*_*i*,1_,…, *Y*_*i*,*m*_ are independent identical observations of the random variable *y_i_* at location *i*. In addition, we assume *Y*_0,1:*m*_,…, *Y*_*n*,1:*m*_ are independent but not necessarily identically-distributed. Let *d_i_* be the geographic distance between location of interest 0 and location *i*. The generalized GWR likelihood is a locally-weighted likelihood:

(1.2) $$p\left(Y_{0:n,1:m}|\theta_{0:n},\varphi_{0}\right)=p\left(Y_{0,1:m}|\theta_{0},\varphi_{0}\right)\prod_{i=1}^{n}p{\left(Y_{i,1:m}|\theta_{i},\varphi_{0}\right)}^{W\left(d_{i},\eta \right)},$$

with coefficient *φ*_0_ and where *W*(*d_i_*, *η*) is a geographically weighted kernel, with band-width *η*, determined by the distance. Following the first law of geography, geographically weighted kernels gradually decrease to 0 as the distance *d_i_* increases. One popular choice of weighted kernel is a Gaussian kernel (Brunsdon et al., 1996)

(1.3) $$W\left(d_{i},\eta \right)=\text{exp}\left(-\frac{d_{i}^{2}}{\eta^{2}}\right),$$

where *η* is a geographical bandwidth which regulates the kernel size.

Inference for GWR models has usually been conducted in a frequentist framework, but a Bayesian extension of the GWR model would allow introduction of prior information, and also simplify situations where the covariance of the estimator is not easily obtainable. However, Bayesian inference for general GWR models is not immediately clear since, (1.2) is not in general a proper probability density if the power terms are not 1. Hence, Bayes’ theorem does not apply. In the special case of a Gaussian likelihood, *W*(*d_i_*, *η*) can be viewed as a scale parameter of *θ_i_* and thus we obtain a proper probability density. This special case has previously been considered, allowing inference for Gaussian GWR models within a standard Bayesian framework (Subedi et al., 2018; Ma et al., 2020). However, a Bayesian extension for a broader distribution family is unclear, and to the best of our knowledge, no previous papers have considered this problem.

In this article, we extend the generalized GWR model to the Bayesian framework and justify its usage. Observe that (1.2), ignoring the power terms, treats data sampled from neighbouring locations *i*, *i* ≠ 0, as if they share the same relationship with covariate *X_i_* as data sampled from the location of interest *i* = 0. This inevitably leads to the problem of misspecification since *φ_i_* ≠ *φ*_0_ due to the spatial non-stationarity. The degree of misspecification depends on the total variation of *φ_i_*. This observation suggests that the essence of the Bayesian GWR model is dealing with misspecification due to incorporating extra observations from neighbouring locations and inspired us to draw ideas from the literature considering partial misspecification of Bayesian models and the modularized Bayesian analysis (Liu et al., 2009). The model involves a geographically powered posterior, with the power term being a deterministic functional form of the geographical distance. The contribution from each location to the inference of the parameter of interest is manipulated through a geographical bandwidth in the power term and we discuss the optimal selection of this bandwidth so that the negative impact from misspecification and positive impact from extra observations are well balanced. We show some theoretical properties of the model and outline the algorithm.

## 2 Robust Bayesian Inference and Modularization

Several attractive properties of Bayesian inference rely on the correct specification of the model. However, it is generally impossible to ensure the correct specification of a complete Bayesian model. Here, we adopt the M-closed view that a model is correctly specified if the true data generating process $\overset{ˇ}{p}(Y)$ is exactly equal to a parametric distribution *p*_*ψ*0_(*Y*|*ψ*_0_), given parameters *ψ*_0_ ∈ Ψ, which is subsequently referred as the likelihood (Bissiri et al., 2016). Misspecification might exist in all aspects of the model, or in only a few components (or modules in the terminology of Liu et al., 2009) of the model.

In the case of all aspects of the model being misspecified, modification of the conventional Bayesian model is required to improve the robustness of the model. One approach is to raise the likelihood to a power term and regard its logarithm as a loss function (Friel and Pettitt, 2008; Bissiri et al., 2016; Holmes and Walker, 2017), to obtain a weighted likelihood similar to the generalized GWR in (1.2):

(2.1) $$p_{\text{pow},\eta }\left(\psi |Y\right)\propto p_{\psi }{\left(Y|\psi \right)}^{\eta }\pi \left(\psi \right).$$

This is called the power posterior or fractional posterior, with power *η*. While weighted likelihoods have a long history in frequentist statistics (e.g., Cai et al., 2000; Markatou, 2000; Hu and Zidek, 2002; Biswas et al., 2015), it is only recently that justification of their usage in Bayesian statistics has been studied. One interpretation of the power term is that it adjusts the sample size with a multiplier *η* (Miller and Dunson, 2019). Another interpretation is that it is equivalent to a data-dependent prior (Martin et al., 2017). Miller and Dunson (2019) further argue that (2.1) approximates $p\left(\psi |D_{KL}\left(p_{\psi }\left(\cdot |\psi \right),\hat{p}\left(\cdot \right)\right)<R\right)$ under mild conditions, where the Kullback-Leibler (KL) divergence $D_{KL}\left(\hat{p}\left(\cdot \right),p_{\psi }\left(\cdot |\psi \right)\right)=\int \hat{p}\left(y\right)\text{log}\left(\hat{p}\left(y\right)/p_{\psi }\left(y|\psi \right)\right)dy$ and *R* is determined by the number of samples and the power *η*. The contraction of the power posterior is shown by Bhattacharya et al. (2019). These papers suggest that, in the case of a M-open view, where the true data generating process does not belong to the parametric distributions termed as likelihood, inference can proceed by looking for parameters whose likelihood approximates the true data generating process. In addition, an appropriate choice of *η* can accommodate this departure of misspecified *p_ψ_* (*Y*|*ψ*) from the truth $\overset{ˇ}{p}(Y)$ and the model is robust (Miller and Dunson, 2019). Importantly, the power *η* controls the relative credence given to the observed data and the prior; consequently it is not deemed as a parameter. Therefore, a prior is not assigned for *η* and it is not updated via Bayes theorem.

**Figure 1:**
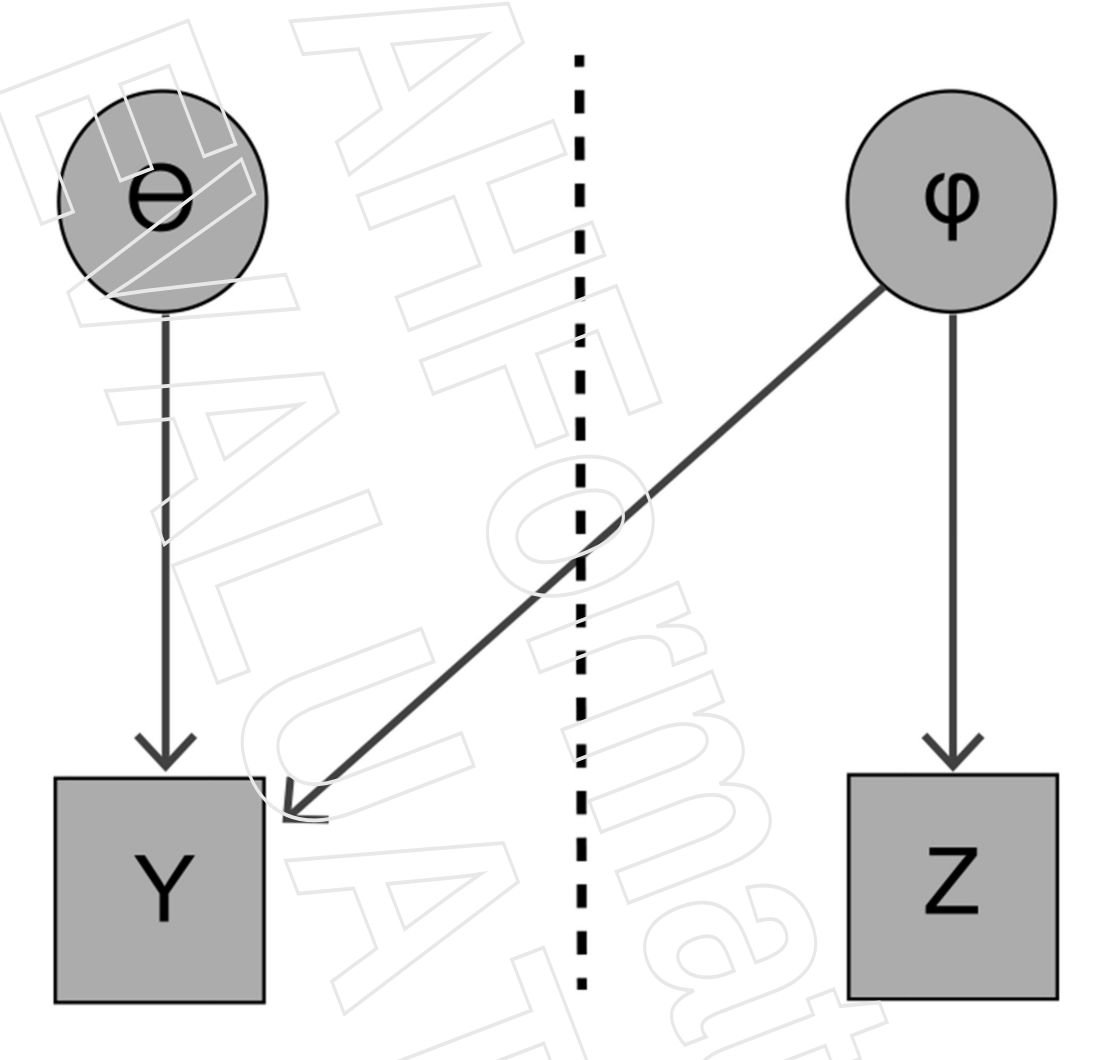
DAG representation of a two module model. The modules are separated by a dashed line.

In the case of partial misspecification, misspecification of even a single module can cause incorrect estimation of other modules, even if these modules are correctly specified (Plummer, 2015; Liu and Goudie, 2022). Consider the two module model illustrated in Figure 1, with likelihood terms *p*(*Y*|*θ*, *φ*) and *p*(*Z*|*φ*), and prior terms *π*(*θ*) and *π*(*φ*). The posterior distribution, with parameters of interest *ψ* = (*θ*, *φ*), is

$$p(\psi |Y,Z)=p(\theta |Y,\varphi )p(\varphi |Y,Z)=\frac{p(Y|\theta ,\varphi )\pi (\theta )}{p(Y|\varphi )}\frac{p(Y|\varphi )p(Z|\varphi )\pi (\varphi )}{p(Y,Z)}.$$

Suppose that the specification of the likelihood for *Y* is suspected to be incorrect. If we wish to prevent *Y* affecting estimation of *φ*, then we can use the cut distribution (Lunn et al., 2009), defined for this model as

$$p_{\text{cut}}(\psi |Y,Z):=p(\theta |Y,\varphi )p(\varphi |Z)=\frac{p(Y|\theta ,\varphi )\pi (\theta )}{p(Y|\varphi )}\frac{p(Z|\varphi )\pi (\varphi )}{p(Z)}.$$

Note that under the cut distribution *φ* depends on only the data *Z*; the data *Y* makes no contribution to the estimation of *φ*. This is called “cutting the feedback” (Lunn et al., 2009). This model has been used for Bayesian propensity scores (e.g., McCandless et al., 2010; Kaplan and Chen, 2012; Zigler and Dominici, 2014) where feedback from the outcome module to the propensity score module should be removed (Rubin, 2008; Zigler et al., 2013). It has also been used in various other fields (e.g., Blangiardo et al., 2011; Arendt et al., 2012; Frank et al., 2019).

The cut distribution and the standard posterior are two extremes: all information from the suspect module is either removed or retained. However, completely cutting or retaining the feedback from the suspect module might either lose usable information or introduce excessive bias. To control the feedback from the potentially misspecified module, a combination of the power posterior and cut model was recently proposed by Carmona and Nicholls (2020). Their Semi-Modular Inference (SMI) model introduces an auxiliary variable $\tilde{\theta }$, which has the same distribution as *θ*, to regulate the contributions to the estimation of *φ*. Given a prior $\pi \left(\varphi ,\tilde{\theta }\right)$, the SMI distribution of the augmented parameter $\left(\theta ,\tilde{\theta },\varphi \right)$ is

$$p_{\eta }(\theta ,\tilde{\theta },\varphi |Y,Z)=p_{\text{pow},\eta }(\tilde{\theta },\varphi |Y,Z)p(\theta |Y,\varphi ),$$

where

$$p_{\text{pow},\eta }\left(\tilde{\theta },\varphi |Y,Z\right)\propto p\left(Z|\varphi \right)p{\left(Y|\varphi ,\tilde{\theta }\right)}^{\eta }\pi \left(\varphi ,\tilde{\theta }\right)$$

is a power posterior of $\tilde{\theta }$ and *φ*, with power *η*. The SMI distribution of the parameters of interest *ψ* = (*θ*, *φ*) is

$$p_{\eta }(\psi |Y,Z)=\int p_{\eta }(\theta ,\tilde{\theta },\varphi |Y,Z)d\tilde{\theta }.$$

The power *η* controls how much information from the suspect module involving *Y* is used to estimate *φ*.

## 3 Modularized Bayesian Inference for Multiple Modules

### 3.1 Standard Bayesian Posterior and Cut Distribution

**Figure 2:**
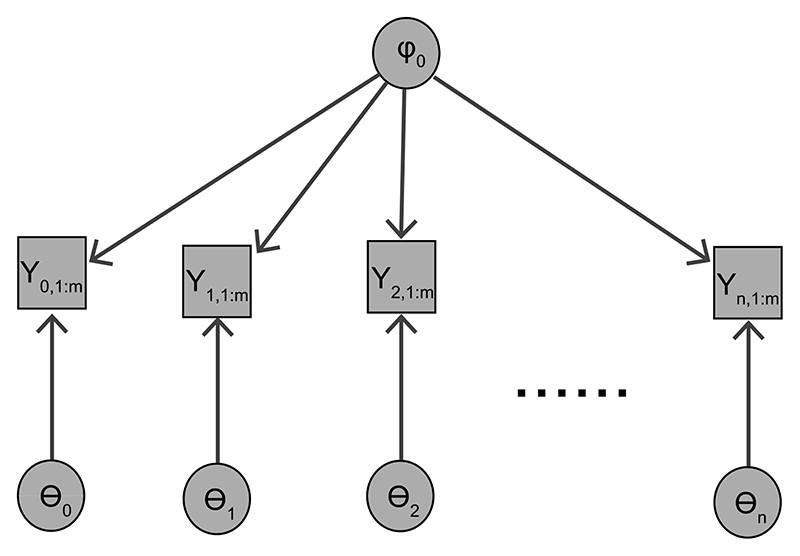
DAG representation when *φ*(*u_i_*, *v_i_*) = *φ*_0_.

To establish notation, first consider the simple case when the spatial coefficient function *φ*(*u_i_*, *v_i_*) = *φ*(*u*_0_, *v*_0_) = *φ*_0_; that is *φ* is constant across the whole geographical space and so we can directly include all data from all locations into the model. Denote the likelihood *p*(*Y*_*i*,1:*m*_|*θ*_*i*_, *φ*_0_) at location *i*, with *i* = 0, 1,…, *n*. The DAG of this model is shown in Figure 2. The joint distribution with an independent prior $\pi \left(\theta_{0:n},\varphi_{0}\right)=\pi \left(\varphi_{0}\right)\prod_{i=0}^{n}\pi \left(\theta_{i}\right)$ is

$$p\left(Y_{0:n,1:m},\theta_{0:n},\varphi_{0}\right)=\pi \left(\theta_{0:n},\varphi_{0}\right)\prod_{i=0}^{n}p\left(Y_{i,1:m}|\theta_{i},\varphi_{0}\right).$$

The following lemma gives the form of the standard Bayesian posterior.

#### Lemma 3.1

*The standard Bayesian posterior is:*

(3.1) $$p\left(\theta_{0:n},\varphi_{0}|Y_{0:n,1:m}\right)=p\left(\theta_{0},\varphi_{0}|Y_{0:n,1:m}\right)\prod_{i=1}^{n}p\left(\theta_{i}|Y_{i,1:m},\varphi_{0}\right).$$

*For proof, see Supplementary Materials* (Liu and Goudie, 2023).

Note that, estimation of (*θ*_0_, *φ*_0_) is influenced by all observations *Y*_0,1:*m*_,…, *Y*_*n*,1:*m*_ as is standard in Bayesian inference: the contribution from any location is equal in the sense that no manipulation of feedback is conducted.

In contrast, consider the case when *φ*(*u_i_*, *v_i_*) is not constant. If we nevertheless include data from location (*u_i_*, *v_i_*), *i* ≠ 0 to estimate the parameter (*θ*_0_, *φ*_0_) and regard *y_i_* ∼ *p*(η|*θ_i_*, *φ*_0_) as module *i*, *i* = 0,1,…, *n*, then the likelihood $\Pi_{i=0}^{n}p\left(Y_{i,1:m}|\theta_{i},\varphi_{0}\right)$ is clearly misspecified since *φ*_0_ ≠ *φ*(*u_i_*, *v_i_*). A straightforward way to handle this misspecification is to remove the influence of these modules on the estimation of *φ*_0_ by using the cut distribution. The cut distribution for this model is:

(3.2) $$p_{\text{cut}}\left(\theta_{0:n},\varphi_{0}|Y_{0:n,1:m}\right):=p\left(\theta_{0},\varphi_{0}|Y_{0,1:m}\right)\prod_{i=1}^{n}p\left(\theta_{i}|Y_{i,1:m},\varphi_{0}\right).$$

Here, estimation of *φ*_0_ depends on only *Y*_0,1:*m*_. Contributions from *Y*_1:*n*,1:*m*_ at other locations are completely removed.

### 3.2 Manipulating the Multiple Feedback and the Bayesian GWR Posterior

Suppose now that *φ*(*u*, *v*) is not constant but is a smooth function with respect to (*u*, *v*) so that closer locations have more similar *φ*. In this case it is inappropriate to treat the misspecification as equally problematic at every location since this may lead to a loss of usable information from the dataset. Instead we propose to manipulate contributions to the estimation of *φ*_0_ from observations *Y*_*i*,1:*m*_ neighbouring the location of interest *i* = 0 by varying amounts. We achieve this by allocating a geographically weighted kernel *W*(*d_i_*, *η*) to the likelihood of *Y*_*i*,1:*m*_ where *d_i_* is the distance between location 0 and location *i*.

**Figure 3:**
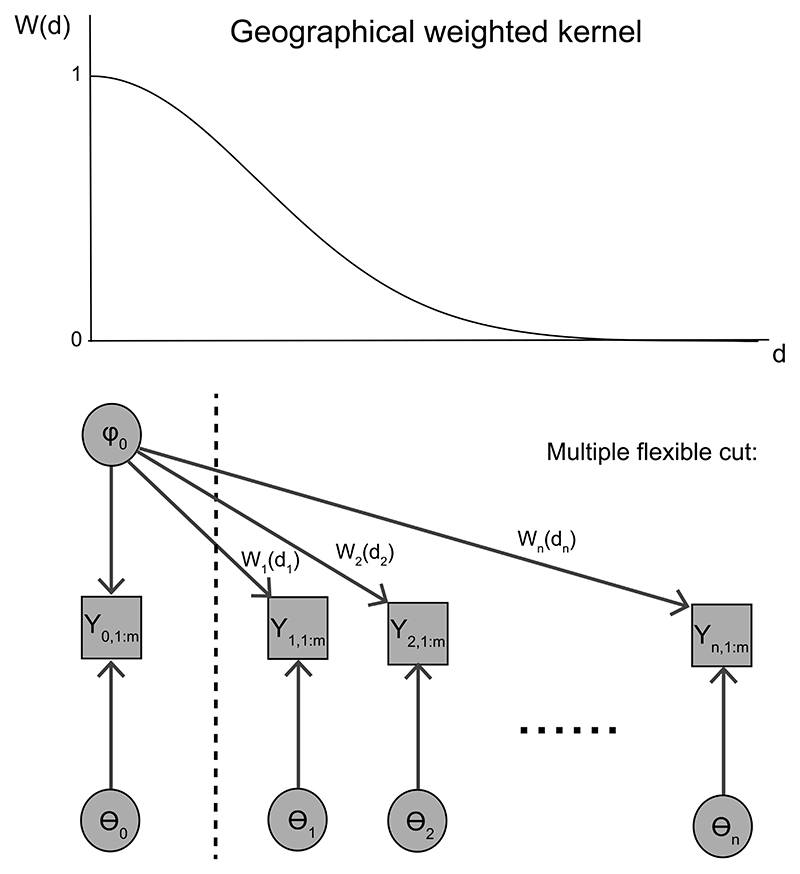
DAG representation when the feedback is manipulated. The *n* + 1 modules (*Y*_*i*,1:*m*_, *φ*_0_, *θ_i_*), *i* = 0,…, *n*, are separated by a dashed line. The location of interest is *i* = 0.

Figure 3 shows a DAG of this model. It can be viewed as a case of manipulating the feedback between *n*+1 modules. Extending Carmona and Nicholls (2020), we introduce an auxiliary variable ${\tilde{\theta }}_{1:n}=\left({\tilde{\theta }}_{1},...,{\tilde{\theta }}_{n}\right)$, which has the same likelihood term as *θ*_1:*n*_. We set an independent prior $\pi \left(\theta_{0},{\tilde{\theta }}_{1:n},\varphi_{0}\right)=\prod_{i=1}^{n}\pi \left({\tilde{\theta }}_{i}\right)\pi \left(\theta_{0}\right)\pi \left(\varphi_{0}\right)$. Then we write

(3.3) $$p_{\eta }\left(\theta_{0:n},{\tilde{\theta }}_{1:n},\varphi_{0}|Y_{0:n,1:m}\right)=p_{\text{pow},\eta }\left(\theta_{0},{\tilde{\theta }}_{1:n},\varphi_{0}|Y_{0:n,1:m}\right)\prod_{i=1}^{n}p\left(\theta_{i}|Y_{i,1:m},\varphi_{0}\right),$$

where

(3.4) $$p_{\text{pow},\eta }\left(\theta_{0},{\tilde{\theta }}_{1:n},\varphi_{0}|Y_{0:n,1:m}\right)\propto p\left(Y_{0,1:m}|\theta_{0},\varphi_{0}\right)\pi \left(\theta_{0},{\tilde{\theta }}_{1:n},\varphi_{0}\right)\prod_{i=1}^{n}p{\left(Y_{i,1:m}|{\tilde{\theta }}_{i},\varphi_{0}\right)}^{W\left(d_{i},\eta \right)}$$

is called the geographically-powered posterior and is used to adjust contributions from observations *Y*_*i*,1:*m*_ by allocating the corresponding weighted kernel *W* (*d_i_*, *η*) to the likelihood $p\left(Y_{i,1:m}|\varphi_{0},{\tilde{\theta }}_{i}\right)$. Note that (3.4) is an extension of the usual power posterior and it contains the GWR locally-weighted likelihood (1.2). Given the geographical bandwidth *η*, the SMI distribution for this multiple module case is

$$p_{\eta }\left(\theta_{0:n},\varphi_{0}|Y_{0:n,1:m}\right)=\int p_{\eta }\left(\theta_{0:n},{\tilde{\theta }}_{1:n},\varphi_{0}|Y_{0:n,1:m}\right)d{\tilde{\theta }}_{1:n}.$$

The Bayesian GWR posterior for the parameters of interest *ψ*_0_ and *θ*_0_ at the location of interest *i* = 0 is

(3.5) $$\begin{array}{lll} p_{\eta }\left(\theta_{0},\varphi_{0}|Y_{0:n,1:n}\right) & = & \int p_{\eta }\left(\theta_{0:n,}\varphi_{0}|Y_{0:n,1:m}\right)d\theta_{1:n}\\ & = & \iint p_{\eta }\left(\theta_{0:n},{\tilde{\theta }}_{1:n},\varphi_{0}|Y_{0:n,1:m}\right)d{\tilde{\theta }}_{1:n}d\theta_{1:n}\\ & = & \int p_{\text{pow},\eta }\left(\theta_{0},{\tilde{\theta }}_{1:n},\varphi_{0}|Y_{0:n,1:m}\right)d{\tilde{\theta }}_{1:n}. \end{array}$$

We call estimation of the parameter of interest (*θ*_0_, *φ*_0_) via (3.5) Bayesian GWR inference. The Bayesian GWR model manipulates the feedback from each of the multiple neighbouring observations through the geographical bandwidth *η*, and reduces to the cut distribution and the standard posterior distribution for certain values for *η*. Specifically, when the variation of *φ*(*u*, *v*) is so large that we are not confident to include neighbouring locations, then *η* → 0 and the estimation of *θ*_0_ and *φ*_0_ only depends on observations *Y*_0,1:*m*_.

$$\underset{\eta \to 0}{\lim}\;p_{\eta }\left(\theta_{0:n},\varphi_{0}|Y_{0:n,1:m}\right)=p\left(\theta_{0},\varphi_{0}|Y_{0,1:m}\right)\prod_{i=1}^{n}p\left(\theta_{i}|Y_{i,1:m},\varphi_{0}\right)=p_{\text{cut}}\left(\theta_{0:n},\varphi_{0}|Y_{0:n,1:m}\right).$$

This is the cut distribution (3.2). In contrast, when the variation of *φ*(*u*, *v*) is so small that we can include observations from all locations, then *η* → ∞ and estimation of *θ*_0_ and *φ*_0_ depends on all observations *Y*_0:*n*,1:*m*_ as in the standard posterior distribution (3.1):

$$\underset{\eta \to \infty }{\lim}p_{\eta }\left(\theta_{0:n},\varphi_{0}|Y_{0:n,1:m}\right)=p\left(\theta_{0},\varphi_{0}|Y_{0:n,1:m}\right)\prod_{i=1}^{n}p\left(\theta_{i}|Y_{i,1:m},\varphi_{0}\right)=p\left(\theta_{0:n},\varphi_{0}|Y_{0:n,1:m}\right).$$

In summary, we propose the Bayesian GWR model for multiple suspect modules for the situation that the geographical weighted kernel (1.3) has a known and deterministic functional form with respect to the geographical coordinates. Since the joint ‘likelihood’ involved in (3.4) is the geographically weighted likelihood widely used in the GWR framework, the essence of the Bayesian GWR model is a particular extension of the SMI model.

### 3.3 Theoretical Analysis

Bayes’ theorem can not be used to justify the proposed geographically-powered posterior because the power likelihood is not a proper probability distribution. Instead, we justify the geographically-powered posterior as a minimizing rule within an information processing framework, thus avoiding the need to appeal to Bayes’ theorem. We also study its property subject to large sample size.

We write the true data generating process for the complete set of observations *Y*_0:*n*,1:*m*_ as

$${\overset{ˇ}{p}}_{0:n,1:m}\left(Y_{0:n,1:m}\right)=\prod_{i=0}^{n}{\overset{ˇ}{p}}_{i,1:m}\left(Y_{i,1:m}\right)=\prod_{i=0}^{n}\prod_{j=1}^{m}{\overset{ˇ}{p}}_{i}\left(Y_{i,j}\right),$$

where ${\overset{ˇ}{p}}_{i}$ is the true generating process at location *i*. Let ${\overset{ˇ}{P}}_{0:n,1:m}$ be the corresponding probability measure. Denoting $\psi =\left(\theta_{0},{\tilde{\theta }}_{1:n},\varphi_{0}\right)\in \Psi$ and *W_i_* = *W*(*d_i_*, *η*) and omitting *η* in *p*_pow,*η*_ for simplicity, the geographically-powered likelihood *p*_pow_(*Y*_0:*n*,1:*m*_|*φ*) for observations *Y*_0:*n*,1:*m*_ is written as (1.2) where *θ_i_* is replaced with $\tilde{\theta }$ for *i* ≠ 0. Let Π be the probability measure of prior distribution. If *p*_pow_(*Y*_0:*n*,1:*m*_):= ∫ *p*_pow_(*Y*_0:*n*,1:*m*_|*ψ*)Π(*dψ*) < ∞, we can re-write the probability measure of geographically-powered posterior (3.4) on any Ψ* ⊂ Ψ in terms of the true data generating processes as

(3.6) $$P_{\text{pow}}\left(\Psi^{*}|Y_{0:n,1:m}\right)=\frac{\int_{\Psi^{*}}\text{exp}\{-r_{0,1:m}\left(\psi \right)-\sum_{i=1}^{n}W_{i}r_{i,1:m}\left(\psi \right)\}\Pi \left(d\psi \right)}{\int_{\Psi }\text{exp}\{-r_{0,1:m}\left(\psi \right)-\sum_{i=1}^{n}W_{i}r_{i,1:m}\left(\psi \right)\}\Pi \left(d\psi \right)},$$

where

$$r_{0,1:m}(\psi )=\text{log}\left\{\frac{{\overset{ˇ}{p}}_{0,1:m}\left(Y_{0,1:m}\right)}{p\left(Y_{0,1:m}|\theta_{0},\varphi_{0}\right)}\right\};r_{i,1:m}(\psi )=\text{log}\left\{\frac{{\overset{ˇ}{p}}_{i,1:m}\left(Y_{i,1:m}\right)}{p\left(Y_{i,1:m}|{\tilde{\theta }}_{i},\varphi_{0}\right)}\right\}i\neq 0.$$

This representation makes it clear that (3.6) is an extension of the Gibbs posterior (Jiang and Tanner,2008), which is also known as the generalized Bayesian posterior (Grünwald and van Ommen,2017); pseudo posterior (Walker and Hjort, 2001; Alquier et al., 2016); and quasi-posterior (Chernozhukov and Hong, 2003; Dunson and Taylor, 2005), which plays an essential role in the study of the PAC-Bayesian inference (e.g., Dalalyan and Tsybakov, 2008; Lever et al., 2013). The Gibbs posterior generalizes the usual Bayesian posterior by defining a prior for the parameter of a loss function, which need not be the negative log-likelihood as used in standard Bayesian inference.

Our model extends the existing Gibbs posterior literature by allowing multiple learning rates (also interpreted as temperatures in thermodynamics (Geman and Geman, 1984)) which correspond to geographically weighted kernels. The loss function (or the statistical risk function) at each location is *W*_*i*_*r*_*i*,1:*m*_(*ψ*), where *W*_0_ = 1. We denote the empirical total loss function *L*_1:*m*_(*ψ*), given the parameter of the model *ψ*,as:

$$L_{1:m}(\psi )=\frac{1}{m}\left(r_{0,1:m}(\psi )+\sum_{i=1}^{n}W_{i}r_{i,1:m}(\psi )\right).$$

Let *F* be a probability measure on the parameter space Ψ which results from processing the information from observations *Y*_0:*n*,1:*m*_ and prior knowledge Π. We aim to show that the geographically-powered posterior *P*_pow_ is the optimal *F* in the sense that *P*_pow_ minimizes an information bound. We first need to construct this information bound. Bhattacharya et al. (2019) provides a PAC-Bayesian type bound for the power posterior. The bound controls a Rényi divergence which characterizes the performance of the power posterior. We now denote the Rényi divergence between two arbitrary distribution *p* and *q*, given an *α* ∈ (0, 1), as:

$$D_{\alpha }(p(\cdot ),q(\cdot ))=\frac{1}{\alpha -1}\text{log}\left(\int p{\left(y\right)}^{\alpha }q{\left(y\right)}^{1-\alpha }dy\right).$$

We have the following theorem that extends the Theorem 3.4 of Bhattacharya et al. (2019) by allowing multiple learning rates.

#### Theorem 3.1

(Weighted Rényi divergence bound). *Given a distribution* *f*(*φ*) *with probability measure* *F*(η) *over parameter space* Ψ, *for any ε* ∈ (0, 1), *the following inequality*

$$\begin{array}{l} \int \sum_{i=1}^{n}\left(1-W_{i}\right)D_{W_{i}}\left(p\left(\cdot |{\tilde{\theta }}_{i},\varphi_{0}\right),{\overset{ˇ}{p}}_{i}(\cdot )\right)F(d\psi )\\ \leq \frac{1}{m}\int \left(r_{0,1:m}(\psi )+\sum_{i=1}^{n}W_{i}r_{i,1:m}(\psi )\right)F(d\psi )+\frac{D_{KL}(f(\cdot ),\pi (\cdot ))}{m}+\frac{1}{m}\text{log}\left(\frac{1}{\varepsilon }\right) \end{array}$$

*holds with* ${\overset{ˇ}{P}}_{0:n,1:m}$ *probability at least* (1 − *ε*).

*For proof, see Supplementary Materials* (Liu and Goudie, 2023).

#### Remark

Theorem 3.1 *leads to the following “information posterior bound” (Zhang, 2006), which holds with* ${\overset{ˇ}{P}}_{0:n,1:m}$ *probability at least* (1 − *ε*).

$$\begin{array}{l} \underset{\psi ∼F}{E}\left\{-\text{log}\left(\underset{Y_{0:n,1:m}∼{\overset{ˇ}{P}}_{0:n,1:m}}{E}\text{exp}\left(-L_{1:m}(\psi )\right)\right)\right\}\\ \leq \underset{\psi ∼F}{E}L_{1:m}(\psi )+\frac{D_{KL}(f(\cdot ),\pi (\cdot ))}{m}+\frac{1}{m}\text{log}\left(\frac{1}{\varepsilon }\right). \end{array}$$

*For proof, see Supplementary Materials* (Liu and Goudie, 2023).

Given a distribution *F* which results from an information processing rule, the Remark states that the negative logarithm of the expected exponential of the negative loss is controlled by the empirical loss from the usage of *F* and an additional penalty on the discrepancy between *F* and the prior Π. Zhang (2006) proposed an approach called “Information Risk Minimization” which selects *F* by minimizing the right hand side of the information posterior bound. Note that, although the bound involves *ε*, the inequality holds for any *ε* ∈ (0, 1). Hence, the selection of *F* is not affected by *ε*. Similarly, the true data generating process drops out since it does not involve *F*. To apply this approach, it is equivalent to find a *F* that minimizes the following criterion function

$$\begin{array}{c} M_{m}(f(\psi ))=D_{KL}(f(\cdot ),\pi (\cdot ))-\int f(\psi )\text{log}\left(p\left(Y_{0,1:m}|\theta_{0},\varphi_{0}\right)\right)d\psi \\ -\sum_{i=1}^{n}W_{i}\int f(\psi )\text{log}\left(p\left(Y_{i,1:m}|{\tilde{\theta }}_{i},\varphi_{0}\right)\right)d\psi . \end{array}$$

Note that the “Information Risk Minimization” used here can be regarded as a modified “Information Conservation Principle” (Zellner, 1988). This principle states that an optimal information processing rule has equal input information *I*_in_, which consists the information processing (i.e., prior knowledge, observations and model), and output information *I*_out_. In our setting, for the probability measure *F*, the input information *I*_in_ is:

$$\begin{array}{c} I_{\text{in}}:=\int \text{log}(\pi (\psi ))F(d\psi )+\int \text{log}\left(p_{\text{pow}}\left(Y_{0:n,1:m}|\psi \right)\right)F(d\psi )\\ =\int \text{log}(\pi (\psi ))F(d\psi )+\int \text{log}\left(p\left(Y_{0,1:m}|\theta_{0},\varphi_{0}\right)\right)F(d\psi )\\ +W_{i}\sum_{i=1}^{n}\int \text{log}\left(p\left(Y_{i,1:m}|{\tilde{\theta }}_{i},\varphi_{0}\right)\right)F(d\psi ). \end{array}$$

Note that in contrast to the original input information discussed in Zellner (1988), the input information from each geographical location is manipulated by the geographically weighted kernel. The output information *I*_out_ is:

$$I_{\text{out}}:=\int \text{log}(f(\psi ))F(d\psi )+\int \text{log}\left(p_{\text{pow}}\left(Y_{0:n,1:m}\right)\right)F(d\psi ).$$

Now we present the following theorem which justifies the use of the geographically-powered posterior (3.6) as the form of probability distribution that statistically learns information from the observations and the prior knowledge while minimising the loss.

#### Theorem 3.2

(Justification). *If* *p_pow_*(*Y*_0:*n*,1:*m*_) := *∫* *p*(*Y*_0:*n*,1:*m*_|*ψ*)*π*(*ψ*)*dψ* < ∞, *the geographical ly-powered posterior* *p_pow_*(*ψ*|*Y*_0:*n*,1:*m*_) *minimizes the criterion function* *M_m_*(*f*(*ψ*)) *with respectto a probability distribution* *f*(*ψ*). *In addition, the geographically-powered posterior results from the optimal information processing rule*.

*For proof, see Supplementary Materials* (Liu and Goudie, 2023).

We now consider the large sample size setting. Let *y*_0:*n*_ = {*y*_0_, *y*_1_,…, *y_n_*} be random variables corresponding to a single observation at each location and *y*_0:*n*_ ~ $P=\prod_{i=1}^{n}{\overset{ˇ}{P}}_{i}$. Although the GWR model is less necessary in the large sample size setting (since effective statistical inference can be conducted separately at each location), we wish to show that the posterior predictive distribution $p\left(y_{i}|{\tilde{\theta }}_{i},\varphi_{0}\right)$, where $\left({\tilde{\theta }}_{i},\varphi_{0}\right)$ ∼ *P*_pow_, approaches the truth ${\overset{ˇ}{p}}_{i}$ at each location *i* = 0, 1,…, *n* when the degree of the partial misspecification varies across the geographical space. Denote the expected total loss function *L*(*ψ*), given the parameter of the model *ψ*, as:

$$L(\psi )=\underset{y_{0:n}∼\overset{ˇ}{P}}{E}\left\{\text{log}\left(\frac{{\overset{ˇ}{p}}_{0}\left(y_{0}\right)}{p\left(y_{0}|\theta_{0},\varphi_{0}\right)}\right)+\sum_{i=1}^{n}W_{i}\text{log}\left(\frac{{\overset{ˇ}{p}}_{i}\left(y_{i}\right)}{p\left(y_{i}|{\tilde{\theta }}_{i},\varphi_{0}\right)}\right)\right\}.$$

We present the following theorem.

#### Theorem 3.3

(Consistency). *Given a finite number of observations, the geographically-powered posterior* *P_pow_* *minimizes*

$$\underset{\psi ∼P_{\text{pow}}}{E}\left(L_{1:m}(\psi )\right)+m^{-1}D_{KL}\left(p_{\text{pow}}\left(\cdot |Y_{0:n,1:m}\right),\pi (\cdot )\right).$$

*When the sample size* *m* → ∞ *at all locations and suppose that the limit of the geographically-powered posterior* $P_{pow}^{\left(\infty \right)}:={\text{lim}}_{m\to \infty }P_{pow}$ *exists, then* $p_{pow}^{\left(\infty \right)}$ *puts all its mass at* $\psi^{*}=\left(\theta_{0}^{*},{\tilde{\theta }}_{1:n}^{*},\varphi_{0}^{*}\right)$ *which minimizes the expected total loss function (a geographically weighted combination of Kullback–Leibler divergences):*

$$\begin{array}{lll} \psi^{*} & = & \underset{\psi =\left(\theta_{0},{\tilde{\theta }}_{1:n},\varphi_{0}\right)}{\text{arg}\;\text{min}}L\left(\psi \right)\\ & = & \underset{\psi =\left(\theta_{0},{\bar{\theta }}_{1:n},\varphi_{0}\right)}{\text{arg}\;\text{min}}D_{KL}\left({\overset{ˇ}{p}}_{0}(\cdot ),p\left(\cdot |\theta_{0},\varphi_{0}\right)\right)+\sum_{i=1}^{n}W_{i}D_{KL}\left({\overset{ˇ}{p}}_{i}(\cdot ),p\left(\cdot |{\tilde{\theta }}_{i},\varphi_{0}\right)\right). \end{array}$$

*For proof, see Supplementary Materials* (Liu and Goudie, 2023).

Although partial misspecification remains and predictions drawn from the model will not follow the true data generating process, Theorem 3.3 states that the geographically-powered posterior draws predictions that balance minimizing the empirical total loss function and the discrepancy between posterior and prior knowledge. When the sample size increases, the model acts similarly to a standard Bayesian model by learning more from observations. In the limit of infinite sample size, the model provides a prediction that is closest to the true data generating process. Note that, although the model draws predictions close to the truth, more priority is assigned to locations close to the location of interest, and so we cannot use a single Bayesian GWR model when inference is needed for multiple locations. Instead, separate models should be used at each location of interest.

## 4 Inference for Multiple Locations and Bandwidth Selection

### 4.1 Predictive Performance of One Bayesian GWR Model

In Section 3, we considered the setting when there is a single location of interest. We now consider inference for multiple sampling locations when all locations are of interest. This is done by using separate Bayesian GWR models for each location while assuming the same geographical bandwidth for all models. We give the following definition which generalizes the Bayesian GWR model by relaxing the location of interest.

#### Definition 4.1

*Consider observations* *Y*_*i*,1:*m*_ *sampled from location* *i* *with coordinate* (*u_i_*, *v_i_*), *i* = 0,…, *n;* *a bandwidth* *η* *and a specific geographical coordinate* (*u*, *v*) *that we call the geographical centre. Define the Bayesian GWR model* *M* = ((*u*, *v*), *η*) *with parameter* *ψ_M_* = (*θ*_*M*,0:*n*_, *ψ_M_*) *to be the SMI model with distribution*

(4.1) $$p_{M}\left(\psi_{M}|Y_{0:n,1:m}\right)=\int p_{M}\left(\psi_{M},{\tilde{\theta }}_{M,0:n}|Y_{0:n,1:m}\right)d{\tilde{\theta }}_{M,0:n},$$

where ${\tilde{\theta }}_{M,1:n}$ *is the auxiliary variable for model M and*

(4.2) $$\begin{array}{ll} p_{M}\left(\psi_{M},{\tilde{\theta }}_{M,0:n}|Y_{0:n,1:m}\right)\propto & \pi \left({\tilde{\theta }}_{M,0:n},\varphi_{M}\right)\\ & \times \prod_{i=0}^{n}p{\left(Y_{i,1:m}|{\tilde{\theta }}_{M,i},\varphi_{M}\right)}^{W\left(d_{i},\eta \right)}\prod_{i=0}^{n}p\left(\theta_{M,i}|Y_{i,1:m},\varphi_{M}\right) \end{array}$$

*where* *d_i_* *is the geographical distance between location* (*u_i_*, *v_i_*) *and the geographical centre* (*u*, *v*). *In the special case when the geographical centre is one of the sampling locations, which we assume without loss of generality to be* (*u*_0_, *v*_0_), *then* (4.1) *and* (4.2) *reduce to* (3.3) *and* (3.4).

To measure the predictive performance of a model *M* for, for example, a new observation $Y_{i}^{*}$ from location *i* with true generating process ${\overset{ˇ}{p}}_{i}\left(Y_{i}^{*}\right)$, we use the Kullback-Leibler (KL) divergence. This is achieved by looking at the expected log pointwise predictive density (Gelman et al., 2014; Jacob et al., 2017), which is essentially a constant term minus the KL divergence, and is defined as

(4.3) $${\text{elpd}}_{\left(u_{i},v_{i}\right)}(M):=\int {\overset{ˇ}{p}}_{i}\left(Y_{i}^{*}\right)\text{log}\left(p_{M}\left(Y_{i}^{*}|Y_{0:n,1:m}\right)\right)dY_{i}^{*},$$

where the predictive distribution $p_{M}\left(Y_{i}^{*}|Y_{0:n,1:m}\right)$ is defined as

$$\begin{array}{ll} p_{M}\left(Y_{i}^{*}|Y_{0:n,1:m}\right): & =\int p\left(Y_{i}^{*}|\theta_{M,i},\varphi_{M}\right)p_{M}\left(\psi_{M}|Y_{0:n,1:m}\right)d\psi_{M}\\ & =\int p\left(Y_{i}^{*}|\theta_{M,i},\varphi_{M}\right)p_{M}\left(\theta_{M,i},\varphi_{M}|Y_{0:n,1:m}\right)d\theta_{M,i}d\varphi_{M}. \end{array}$$

Here, we denote *p_M_*(*θ*_*M*,*i*_, *φ*_*M*_|*Y*_0:*n*,1:*m*_) := *∫* *p_M_*(*ψ_M_*|*Y*_0:*n*,1:*m*_)*dθ*_*M*,-*i*_, where we define *θ*_*M*,-*i*_ = (*θ*_*M*,0_,…, *θ*_*M*,*i*-1_, *θ*_*M*,*i*+1_,…, *θ*_*M*, *n*_).

### 4.2 Inference for Multiple Locations

Having defined the measure of predictive performance for one Bayesian GWR model, we are ready to extend it to infer multiple locations by setting and tuning multiple Bayesian GWR models. The following assumption can be viewed as a rephrasing of the first law of geography (Tobler, 1970), since for an arbitrary location of interest *i*, observations from closer locations contribute more to the estimation of the shared parameter *φ* when the geographical centre is exactly equal to the location of interest.

#### Assumption 4.1

*For any fixed geographical bandwidth* *η* *and specific location with geographical coordinates* (*u_k_*, *v_k_*), *elpd*_(*u_k_*, *v_k_*)_(*M*) *is maximized when the geographical centre* (*u*, *v*) = (*u_k_*, *v_k_*). *That is:*

$$\left(\left(u_{k},v_{k}\right),\eta \right)=\underset{M}{\text{arg}\;\text{max}}\;elpd_{\left(u_{k},v_{k}\right)}(M),\forall \eta .$$

We define the space of Bayesian GWR models 𝓜 = {*M* = ((*u*, *v*), *η*) : *η* > 0}. The following assumption assumes inferences from multiple models are independent.

#### Assumption 4.2

*Given a dataset* *Y*_0:*n*,1:*m*_ *and Bayesian GWR models* *M_s_* ∈ *M*, *s* = 1,…, *S*, *we have the joint Bayesian GWR posterior*

$$p\left(\psi_{M_{1}},\ldots ,\psi_{M_{S}}|Y_{0:n,1:m}\right)=\prod_{s=1}^{S}p_{M_{s}}\left(\psi_{M_{s}}|Y_{0:n,1:m}\right).$$

We are now ready to extend inference to multiple locations. Given a set of Bayesian GWR models *M* = (*M*_0_,…, *M_n_*), one for each geographic sampling location, all with identical geographical bandwidth *η*, we define the expected log pointwise predictive density for new observations $Y_{0:n}^{*}=\left(Y_{0}^{*},...,Y_{n}^{*}\right)$ with each single observation $Y_{i}^{*}$ from location *i* as

$$\text{elpd}(M)=\int \text{log}\left(p\left(Y_{0:n}^{*}|Y_{0:n,1:m}\right)\right)\prod_{i=0}^{n}{\overset{ˇ}{p}}_{i}\left(Y_{i}^{*}\right)dY_{0:n}^{*},$$

where

$$p\left(Y_{0:n}^{*}|Y_{0:n,1:m}\right)=\int p\left(Y_{0:n}^{*}|\psi_{M_{0}},\ldots ,\psi_{M_{n}}\right)p\left(\psi_{M_{0}},\ldots ,\psi_{M_{n}}|Y_{0:n,1:m}\right)d\psi_{M_{0}}\cdots d\psi_{M_{n}}.$$

We then present the following theorem to select the optimal bandwidth.

#### Theorem 4.1

(Bandwidth selection). *Given* Assumption 4.1 *and* 4.2, *for observations* *Y*_0:*n*,1:*m*_ *sampled from locations* *i* *with coordinates* (*u_i_*, *v_i_*), *i* = 0,…, *n*, *the optimal combination of* *n*+1 *separate Bayesian GWR models* *M* = (*M*_0_,…, *M_n_*) *that maximizes elpd* (*M*), *where each* $M_{i}=\left(\left(u_{i}^{*},v_{i}^{*}\right),\eta^{*}\right)$ *is used for prediction in location i, satisfies*

1. *For all* 0 ≤ *i* ≤ *n*,
  $$\left(u_{i}^{*},v_{i}^{*}\right)=\left(u_{i},v_{i}\right).$$
2. *Redefine* *M_i_*(*η*) = ((*u_i_*, *v_i_*), *η*), *then the optimal bandwidth* η* *maximizes the mean (across all sampling locations) expected log pointwise predictive density.*
  $$\eta^{*}=\underset{\eta }{\text{arg}\;\text{max}}\frac{1}{n+1}\sum_{i=0}^{n}elpd_{\left(u_{i},v_{i}\right)}\left(M_{i}\left(\eta \right)\right).$$

*For proof, see Supplementary Materials* (Liu and Goudie, 2023).

In practice, we do not know the true data generating process ${\overset{ˇ}{p}}_{i}$. Numerous methods (e.g., Gelman et al., 2014) can be applied to approximate (4.3). Here, we adopt cross-validation to estimate elpd_(*u_i_*, *v_i_*)_(*M_i_*) because it measures out-of-sample predictive performance and consequently avoids overestimating elpd. We train the model *M_i_* on all observations from other locations *Y*_*j*,1:*m*_, *j* ≠ *i* and a subset *Y*_*i*,1:*m*’_ of the observations from location *i* (denoted as {*Y*_0:*n*,1:*m*_ \ *Y*_*i*,*m*’+1:*m*_}), and estimate elpd using the test set *Y*_*i*,*m*’+1:*m*_ by

(4.4) $$\begin{array}{l} {\hat{\text{elpd}}}_{\left(u_{i},v_{i}\right)}\left(M_{i}\right)\\ =\frac{1}{m-m\prime }\sum_{j=m\prime +1}^{m}\text{log}\left(\int p\left(Y_{i,j}|\psi_{M_{i}}\right)p_{M_{i}}\left(\psi_{M_{i}}|\left\{Y_{0:n,1:m}\backslash Y_{i,m'+1:m}\right\}\right)d\psi_{M_{i}}\right). \end{array}$$

The integral within (4.4) can be easily approximated by the Monte Carlo samples drawn from the Bayesian GWR posterior. This is summarized in Algorithm 1.

### 4.3 Algorithm and Simplification of Computation

We summarize the algorithm for the Bayesian GWR model when there are *n* + 1 locations. For a set of candidate geographical bandwidths ${\left\{\eta_{r}\right\}}_{r=1}^{R}$, we select the optimal geographical bandwidth *η* using Algorithm 1. In Algorithm 2, samples at each iteration can be drawn by using any standard sampler (e.g., Metropolis-Hastings or Gibbs sampler). The algorithm requires an approximation of the elpd at each location separately. This can be done in parallel to expedite computation. Once the optimal geographical bandwidth *η* has been selected, we refit model with this bandwidth to the whole dataset, as described in Algorithm 2. We provide the code for both algorithms in Python Version 3 (https://github.com/MathBilibili/Bayesian-geographically-weighted-regression).

**Algorithm 1 Selection of geographical bandwidth *η* by cross-validation**

**Require:** A candidate set of geographical bandwidths ${\left\{\eta_{r}\right\}}_{r=1}^{R}$, observations *Y*_0:*n*,1:*m*_ and its corresponding coordinates ${\left\{\left(u_{i},v_{i}\right)\right\}}_{i=0}^{n}$, likelihood *p*(*Y*|*θ*, *φ*), prior *π*(*φ*), *π* ($\tilde{\theta }$) and *π*(*φ*), number of iterations *S*, number *Q* of *k*-fold cross-validation folds.

1: **for** *r* ∈ {1,…,*R*} **do**

2:     **for** *q* ∈ {1,…,*Q*} **do**

3:          Select the test set *Y*_*i*,(*m*’+1:*m*)_, a random 100/*k*% subset of observations at location *i*, and training set *Y*^(*i*)^ = *Y*_0:*n*,1:*m*_ \ *Y*_*i*,(*m*’+1.*m*)_ for location *i*, *i* = 0,…,*n*.

4:          Call Algorithm 2 with Bayesian GWR models *M_i_*(*η_r_*) = ((*u_i_*, *v_i_*), *η_r_*)and location-specific dataset *Y*^(*i*)^, *i* = 0,…,*n*.

5:          Calculate ${\hat{\text{elpd}}}_{\left(u_{i},v_{i}\right)}\left(M_{i}\left(\eta_{r}\right)\right)$ on the test set *Y*_*i*,(*m*’+1:*m*)_ using samples ${\left\{\left(\varphi_{i}^{\left(s\right)},\theta_{i}^{\left(s\right)}\right)\right\}}_{s=1}^{S}$, *i* = 0,…,*n*.

6:          Calculate *q^th^* mean elpd: $\bar{{\text{elpd}}_{q}}\left(\eta_{r}\right)=\frac{1}{n+1}\sum_{i=0}^{n}{\hat{\text{elpd}}}_{\left(u_{i},v_{i}\right)}\left(M_{i}\left(\eta_{r}\right)\right)$.

7:     **end for**

8: **end for**

9: **return** ${\left\{{\left\{\bar{{\text{elpd}}_{q}}\left(\eta_{r}\right)\right\}}_{q=1}^{Q}\right\}}_{r=1}^{R}$.

The computational cost of a Bayesian GWR model for multiple locations is mainly determined by two factors when using a Metropolis-Hasting sampler. The first factor is the number of observations *m* at each location, which clearly determines the number of likelihood evaluations required. In practice, this evaluation normally benefits from vectorization.

**Algorithm 2 Bayesian GWR model for multiple locations**

**Require:** A geographical bandwidth *η*, observations *Y*_0:*n*,1:*m*_ and corresponding coordinates ${\left\{\left(u_{i},v_{i}\right)\right\}}_{i=0}^{n}$, likelihood *p*(*Y*|*θ*, *φ*), prior *π*(*θ*), *π*($\tilde{\theta }$) and *π*(*ψ*), number of iterations *S*.

1: Set Bayesian GWR models *M_i_*(*η*) = ((*u_i_*, *v_i_*), *η*) and location-specific dataset *Y*^(*i*)^, *i* = 0,…,*n*. Note that *Y*^(*i*)^ = *Y*_0:*n*,1:*m*_ if cross-validation is not required.

2: **for** *i* ∈ {0,…,*n*} **do**

3:      Calculate geographically weighted kernels, where the distance is calculated between (*u_j_*, *v_j_*) and geographical centre (*u_i_*, *v_i_*) for *j* = 0,…,*n*.

4:      Draw samples $\left\{\theta_{i}^{\left(s\right)},{\tilde{\theta }}_{-i}^{\left(s\right)},\varphi_{i}^{\left(s\right)}\right\}$ from $p_{\text{pow},\eta }\left(\theta_{i},{\tilde{\theta }}_{-i},\varphi_{i}|Y^{\left(i\right)}\right)$, *s* = 1, …, *S*, according to (3.4), with location of interest *i* and ${\tilde{\theta }}_{-i}=\left({\tilde{\theta }}_{0},\ldots ,{\tilde{\theta }}_{i-1},{\tilde{\theta }}_{i+1},\ldots ,{\tilde{\theta }}_{n}\right)$.

5: **end for**

6: **return** Bayesian **GWR** posterior samples ${\left\{{\left\{\left(\varphi_{i}^{\left(s\right)},\theta_{i}^{\left(s\right)}\right)\right\}}_{s=1}^{S}\right\}}_{i=0}^{n}$.

The other factor is the number of locations *n*. On the one hand, by Assumption 4.2, inference of parameters at each location is conducted using *n* separate Bayesian GWR models, which can be easily parallelized. This can greatly reduce the computation time. On the other hand, when using the geographically weighted kernel (1.3), (3.4) requires the powered likelihood to be evaluated *n* times. When this computational cost is too large, it is possible to reduce the load by disregarding distant locations with only tiny weights. Specifically, inspired by the bi-square weighting function (Brunsdon et al., 1996), a modified truncated Gaussian kernel may be useful:

(4.5) $$W\left(d_{i},\eta \right)=\{\begin{array}{ll} \text{exp}\left(-\frac{d_{i}^{2}}{\eta^{2}}\right) & \text{if}\;\text{exp}\left(-\frac{d_{i}^{2}}{\eta^{2}}\right)>W^{*}\\ 0 & \text{otherwise} \end{array},$$

where W* (e.g., 10^−2^) is a threshold value that controls the degree of exclusion. We want this exclusion to reduce the number of likelihood evaluations needed, while retaining all information from the neighbouring locations. A practical way to check this is by looking at the percentage change of the value of (3.4) between kernels (1.3) and (4.5). If the percentage change is trivial, (4.5) will closely approximate (1.3) but at a much lower computational cost, especially when a small bandwidth *η* is adopted. In summary when adopting kernel (4.5), the computational complexity, in terms of evaluating the likelihood of one observation of one MCMC iteration for one location of interest, is 𝒪(*m* × *n*(W*)), where *n*(W*) is the number of locations for evaluations in (3.4) with threshold W*.

## 5 Simulation

To illustrate our methodology and the influence of the geographical bandwidth, we simulated data on a 40 × 40 regular lattice (*u*, *v*), with *u* = 1,…,40 and *v* = 1,…,40, with geographically varying coefficients *φ* = (*φ*_0_, *φ*_1_(*u*, *v*), *φ*_2_(*u*)) defined as:

$$\begin{array}{lll} \varphi_{0} & = & 3,\\ \varphi_{1}\left(u,v\right) & = & 0.1+0.01\sqrt{u^{2}+v^{2}},\\ \varphi_{2}\left(u\right) & = & 0.05\left(\text{sin}\left(\pi /2+\pi \left(u/20\right)\right)+\text{cos}\left(\pi /2+\pi \left(u/20\right)\right)+4\right). \end{array}$$

We generated the true *θ*(*u*, *v*) ∼ *N*(0.5, 0.01^2^) independently: the resulting *θ*(*u*, *v*) is relatively constant across spatial locations, and its variability is not spatially smooth. With these coefficients, we simulated *m* = 100 independent samples at each location from a negative binomial distribution, with covariates *X* = (*X*_1_, *X*_2_, *X*_3_) where *X*_1_ = 1 and *X*_2_ and *X*_3_ drawn from a uniform distribution **U**(0, 10) and **U**(2, 7). The Supplementary Materials (Liu and Goudie, 2023) contains results when the number of independent samples is reduced to *m* = 50 and *m* = 10.

We then fitted Bayesian GWR model to each location separately and independently using the truncated Gaussian kernel with threshold 10^−2^, with geographical bandwidth *η*. The difference in (3.4) using a truncated and non-truncated Gaussian kernel was less than 10^−5^%, suggesting the truncated kernel closely approximates the non-truncated kernel. To estimate the elpd by cross validation, we excluded half of the samples at the location of interest from the training set. We drew 4×10^3^ iterations for each of 10 independent chains at each location, discarding the first 1 × 10^3^ samples as burn-in. We also fitted the PICAR model (Lee and Haran, 2022) as a reference. The details and settings of the PICAR model can be found in the Supplementary Materials (Liu and Goudie, 2023).

**Figure 4:**
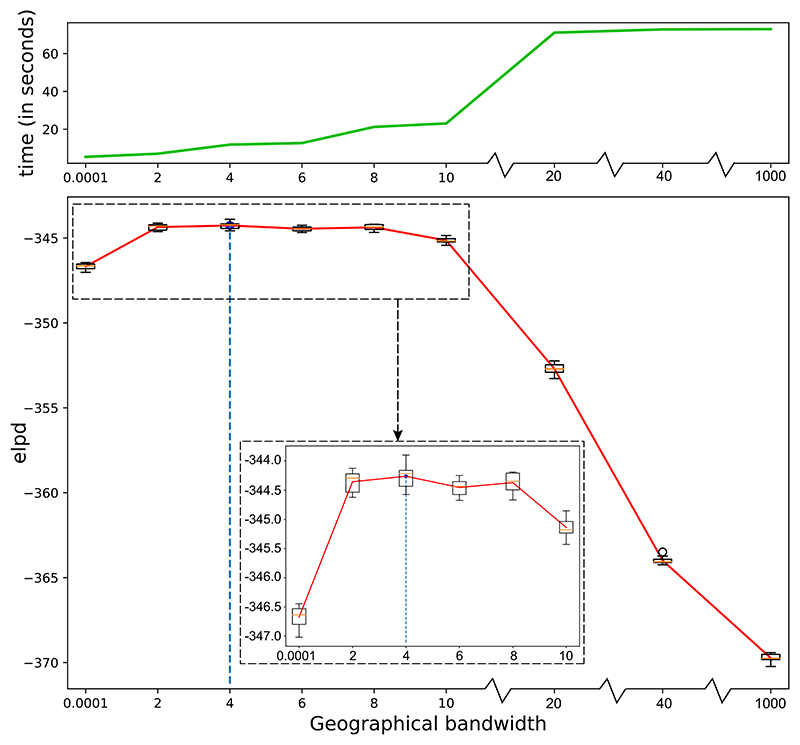
Computational time and elpd against geographical bandwidth. The computational time is calculated based on one MCMC iteration of running Bayesian GWR model for all locations. This is processed in parallel on ten cores of Intel Xeon E7-8860 v3 CPU. Each boxplot represents the elpd estimates from 10 chains across the whole geographic space. The red line is the average elpd estimates across the 10 chains. The blue dashed line indicates the optimal bandwidth. Two black dashed areas are equivalent the inset figure is a zoomed-in version.

To identify the optimal geographical bandwidth *η* for Bayesian GWR, we repeated this process for each of the 9 candidate values *η* = 0.0001, 2, 4, 6, 8, 10, 20, 40, and 1000. Figure 4 shows the computational time and estimated mean expected log pointwise predictive density (mean elpd across space), according to (4.3), for each candidate value. It can be seen that the mean elpd achieves its highest value when the bandwidth is 4, so we will compare results with *η* = 4, *η* = 0.0001 (the smallest candidate, equivalent to using samples only from the geographic centre) and *η* = 1000 (the largest candidate, assuming the least geographic variation).

**Figure 5:**
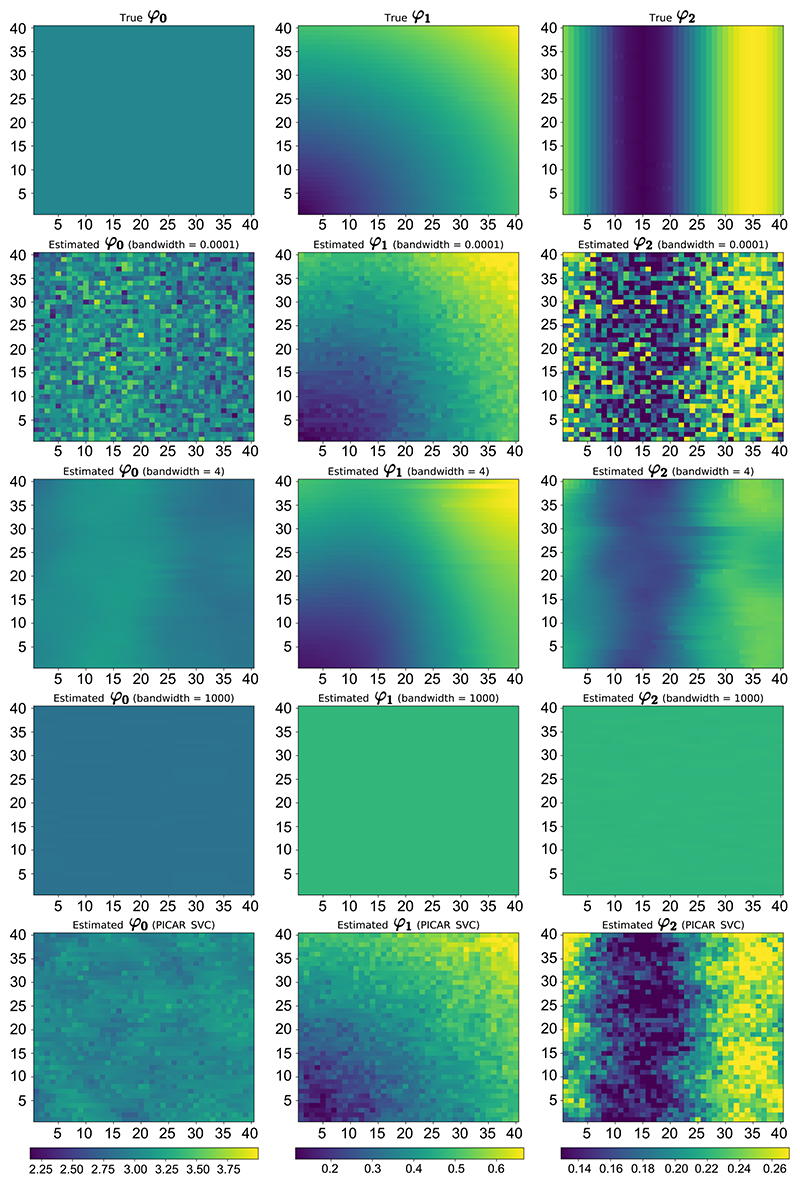
Heatmap of the true values and estimated means for the coefficients *φ*_0_, *φ*_1_ and *φ*_2_, using PICAR and the Bayesian GWR model, with geographic bandwidth *η* = 0 0001, 4 and 1000.

We then ran the model on the complete dataset without excluding any observations. For each location, we ran 10 chains independently for 4 × 10^3^ iterations, discarding the first 1 × 10^3^ samples as burn-in, so that the change of the value of the estimated elpd was smaller than 0.05 (trace plot in Supplementary Materials (Liu and Goudie, 2023)). The true values and estimated means for coefficients (*φ*_0_, *φ*_1_, *φ*_2_) obtained via applying PICAR and Bayesian GWR model, when *η* = 0.0001, 4, 1000, are shown in Figure 5. When *η* = 0.0001, estimation at each location relies almost exclusively on data from that location, so the estimated coefficients vary considerably across spatial locations: the connection between locations is almost completely “cut”. Furthermore, some estimates are extreme because excluding neighbouring samples means only a small number of samples are used by the model. These results reveal the nature of using a small bandwidth in a GWR model, as has also been discussed previously (Guo et al., 2008). In contrast, when *η* = 1000, we can see the estimated coefficients are almost constant across geographic locations, due to the large bandwidth that assumes samples from neighbouring locations are very similar to samples from the location of interest. Finally, the estimates obtained via applying PICAR and Bayesian GWR using the optimal bandwidth *η* = 4 are close to the true values across all geographic locations, although estimates from Bayesian GWR appear to be slightly more smoothed.

**Figure 6:**
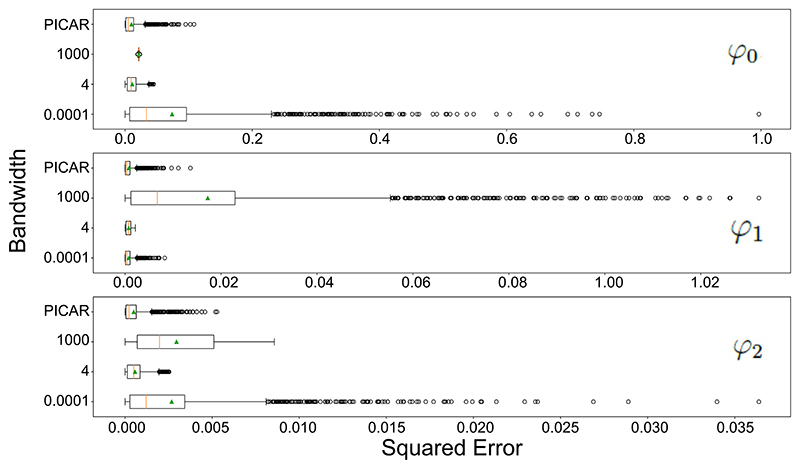
Boxplots of the squared error of the estimated mean coefficients *φ*_0_, *φ*_1_ and *φ*_2_ under the Bayesian GWR model and PICAR model across geographic locations, with geographic bandwidth *η* = 0.0001, 4, and 1000. The orange line and green triangle indicate the median and mean squared error.

Figure 6 shows boxplots of the squared error between the Bayesian GWR and PICAR estimated means and the true values of the three coefficients across all geographic locations. The true *φ*_0_ is constant, therefore a large bandwidth that incorporates more samples will have a lower mean squared error. Hence, the model with *η* = 4 provides good estimation of *φ*_0_. In contrast, the model with *η* = 0.0001 fails to estimate the true value of *φ*_0_ because the sample size at each location is not sufficient to enable precise estimation. Moreover, the model with *η* = 1000 has a significant bias because it incorporates too much information from other locations which have considerably different data generating processes to the location of interest. For *φ*_1_ and *φ*_2_ which do vary geographically, the model with *η* = 1000 as expected performs poorly because the model assumes little geographic variation. The model with *η* = 0.0001 also performs poorly due to the insufficient sample size at each individual location. Overall, the model with the optimal bandwidth *η* = 4 performs the best in mean squared error. When comparing PICAR with Bayesian GWR using the optimal bandwidth, both models give similar results. PICAR on average has slightly lower squared error than our proposed Bayesian GWR model, but the squared error from PICAR appears to be more variable than Bayesian GWR. This is due to the fact that GWR model is an intrinsically smoothing approach. The Supplementary Material (Liu and Goudie, 2023) contains further discussion of the estimation error induced by applying Bayesian GWR with different bandwidths.

## 6 Application to Real Data

It has been shown in epidemiological studies that there is a global variation in the seasonal activity of the influenza virus (e.g., Finkelman et al., 2007; Azziz Baumgartner et al., 2012; Lam et al., 2019). In particular, there are normally clear and consistent influenza epidemic peaks during the winter in the high-latitude regions (Cox and Subbarao, 2000), whereas seasonal transmission patterns are unclear in low-latitude (subtropical/tropical) regions (Viboud et al., 2006; Li et al., 2019). This suggests that transmission and viability of the influenza virus is linked with atmospheric conditions: the regular occurrence of influenza epidemic in temperate regions is largely attributed to the exposure of cold and dry environments (e.g., Lowen et al., 2007; Lowen and Steel, 2014; Deyle et al., 2016; Chong et al., 2020). However, this relationship is weaker in sub-tropical/tropical regions (Tamerius et al., 2013). In this section, we apply the Bayesian GWR model to a human influenza dataset to assess spatial variation in the association between the occurrence of influenza and two major climatic factors (temperature and precipitation). Note that, it has been shown that the relationship between the occurrence of influenza and climatic factors may be temporally varying as well (see Liu et al. (2018)). We ignore these temporal effects here since the extension of GWR models to geographically-temporally weighted regression (GTWR) within a Bayesian framework is not straightforward and requires further study (see Section 7).

We used monthly, country-level human influenza surveillance data between January 2010 and December 2014 from the World Health Organization FluNet (https://www.who.int/tools/flunet). We selected 20 countries of similar size and with relatively comprehensive influenza records. We selected 16 European countries to represent the temperate region (Austria; Belgium; Bosnia and Herzegovina; Croatia; Czech; France; Germany; Hungary; Italy; Luxembourg; Netherlands; Poland; Romania; Slovakia; Slovenia; UK) and 4 South-East Asian countries to represent the tropical region (Cambodia; Laos; Thailand; Vietnam). We used the geographical center coordinates (*u_i_*, *v_i_*), *i* = 1, …, 20 of each country as the geographical coordinates. The dataset contains the number of positive cases *Y*_*i*,*t*_ and total number of tests *N*_*i*,*t*_ in country *i* = 1, …, 20 during month *t*. The temperature *X*_*i*1,*t*_ (degrees Celsius) and amount of precipitation *X*_*i*2,*t*_ (mm/month) during month *t* in country *i* were obtained from CRUCY (Harris et al., 2014).

**Figure 7:**
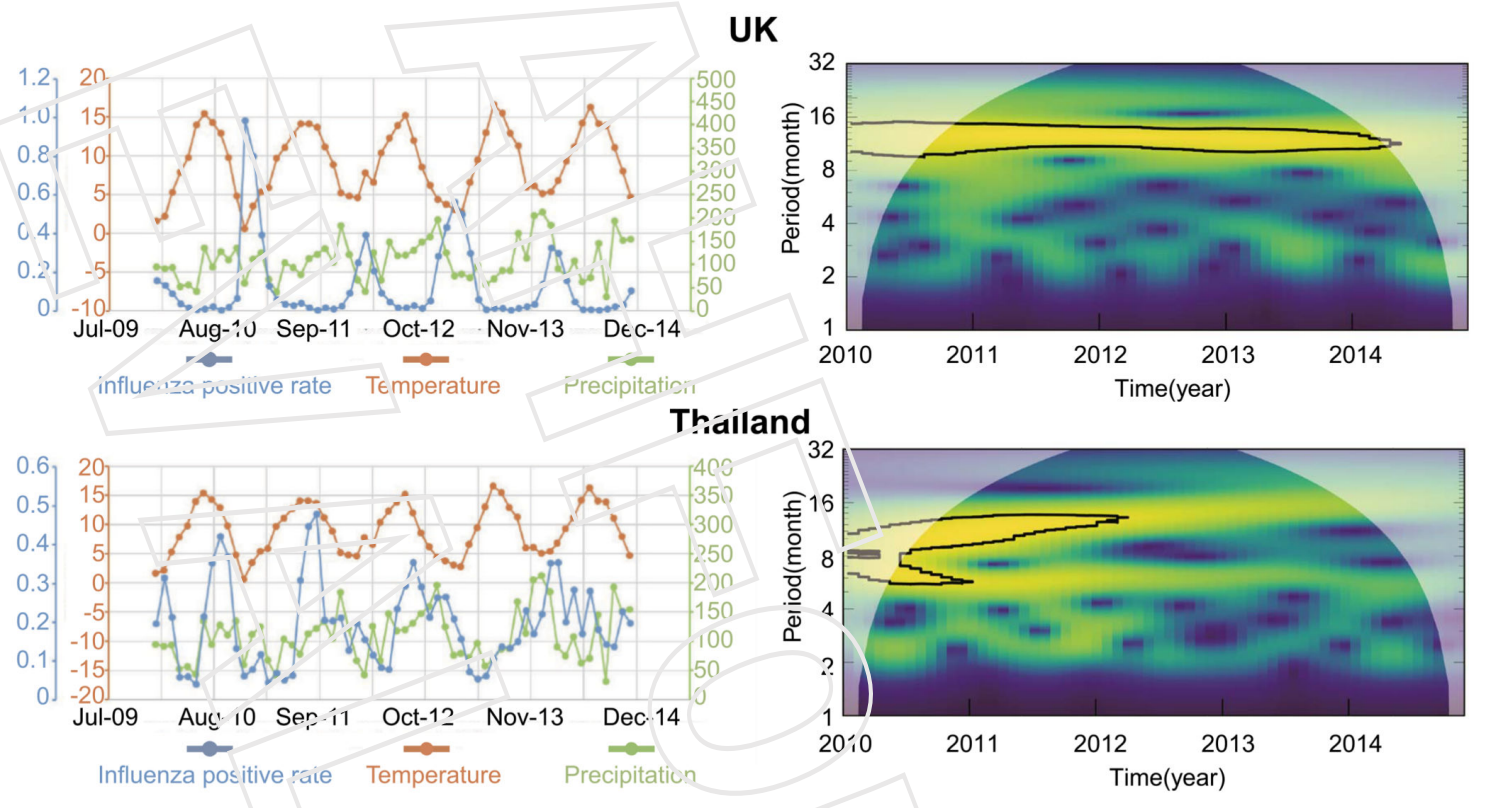
Association between influenza, temperature and precipitation in the UK and Thailand. Left panel: Monthly influenza positivity rate, temperature (degrees Celsius) and precipitation (mm/month). Right panel: Wavelet power spectrum (absolute square of the wavelet transform) of the influenza activity. The black line surrounds the significant area (p-value < 0.01), where the power spectrum is significantly large than the power spectrum of random noise.

The countries we included show distinct patterns of influenza activities. Figure 7 shows, for the UK and Thailand, the monthly influenza positivity rate, temperature, precipitation and the corresponding wavelet analysis of the periodicity of influenza activity. In the UK, we can observe that the peak of influenza activity is consistent with the winter season in the UK and a clear negative correlation can be observed between influenza positivity rate and temperature. The relationship visually appears less strong for precipitation. In contrast, in Thailand influenza has a more variable peak time and the relationship with temperature and precipitation is not clear. To further quantify the distinct seasonality of influenza activities between two countries for better understanding of the underlying geographical difference, we conducted a separate (exploratory) wavelet analysis using WaveletComp in R. This decomposes the influenza time series into numerous wavelets, each with a distinct frequency. The degree to which influenza follows a particular periodicity can be assessed by the magnitude of the corresponding wavelet. This reveals clear evidence of periodicity of between 10–15 months in all years in the UK, whereas there is no consistent periodicity in Thailand (Figure 7). This highlights the potential geographical variation of the influenza activities, suggesting a GWR model is appropriate.

In our Bayesian GWR model, we assumed that the number of positive cases *Y*_*i*,*t*_ follows a negative binomial distribution, as in (1.1), except that the total number of tests *N*_*i*,*t*_ was embedded into the link function and spherical distance was calculated using the haversine formula. The mean and variance of *Y*_*i*,*t*_ are:

$$\begin{array}{l} \;E\left(Y_{i,t}|X_{i1,t},X_{i2,t}\right)=\text{exp}\left(\text{log}\left(N_{i,t}\right)+\varphi_{0}\left(u_{i},v_{i}\right)+\varphi_{1}\left(u_{i},v_{i}\right)X_{i1,t}+\varphi_{2}\left(u_{i},v_{i}\right)X_{i2,t}\right),\\ \text{Var}\left(Y_{i,t}|X_{i1,t},X_{i2,t}\right)=E\left(Y_{i,t}|X_{i1,t},X_{i2,t}\right)+\theta_{i}E^{2}\left(Y_{i,t}|X_{i1,t},X_{i2,t}\right). \end{array}$$

**Figure 8:**
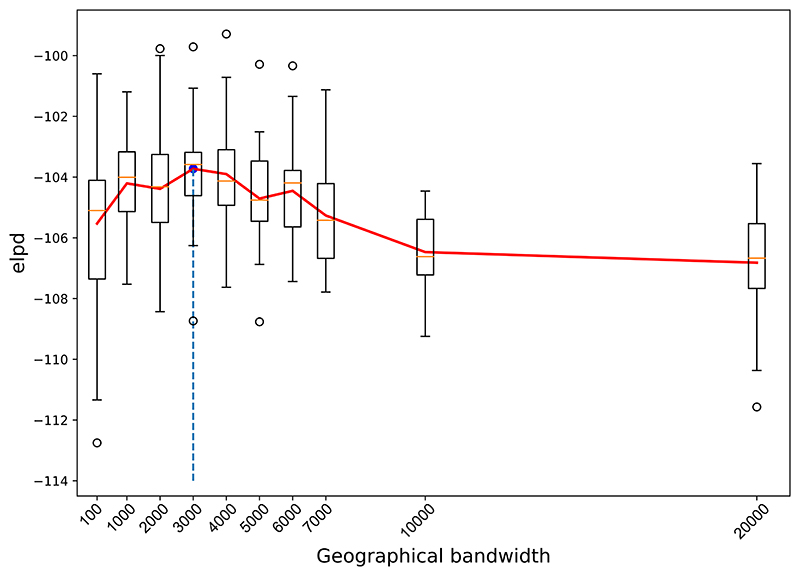
ELPD against geographical bandwidth. Each boxplot represents the elpd estimates from 30 chains across 20 countries. The red line is the average elpd estimates across the 30 chains. The blue dashed line indicates the optimal bandwidth.

We considered each of the 20 countries separately, with each of the following geographical bandwidths *η* = 100, 1000, 2000, 3000, 4000, 5000, 6000, 7000, 10000 and 20000 (kilometres) for a Gaussian kernel. These choices of bandwidth cover a broad range of different assumptions regarding the impact of neighbouring countries. For each country, we randomly left-out 50% of the observations to use as a test set. We ran 30 independent MCMC chains, and after discarding the first 3 × 10^3^ samples, we drew 10^4^ samples from the Bayesian GWR posterior. Figure 8 shows the estimated elpd for each bandwidth across the whole space, suggesting that the optimal choice of the bandwidth from the candidate set is 3000. This bandwidth indicates that there is spatial variation of the underlying association across the countries we selected. Note that, the range of 3000 kilometres roughly spans either Europe or South-East Asia but not both, meaning that spatial non-stationarity was detected between these two regions but the spatial non-stationarity is not significant within the two regions.

**Figure 9:**
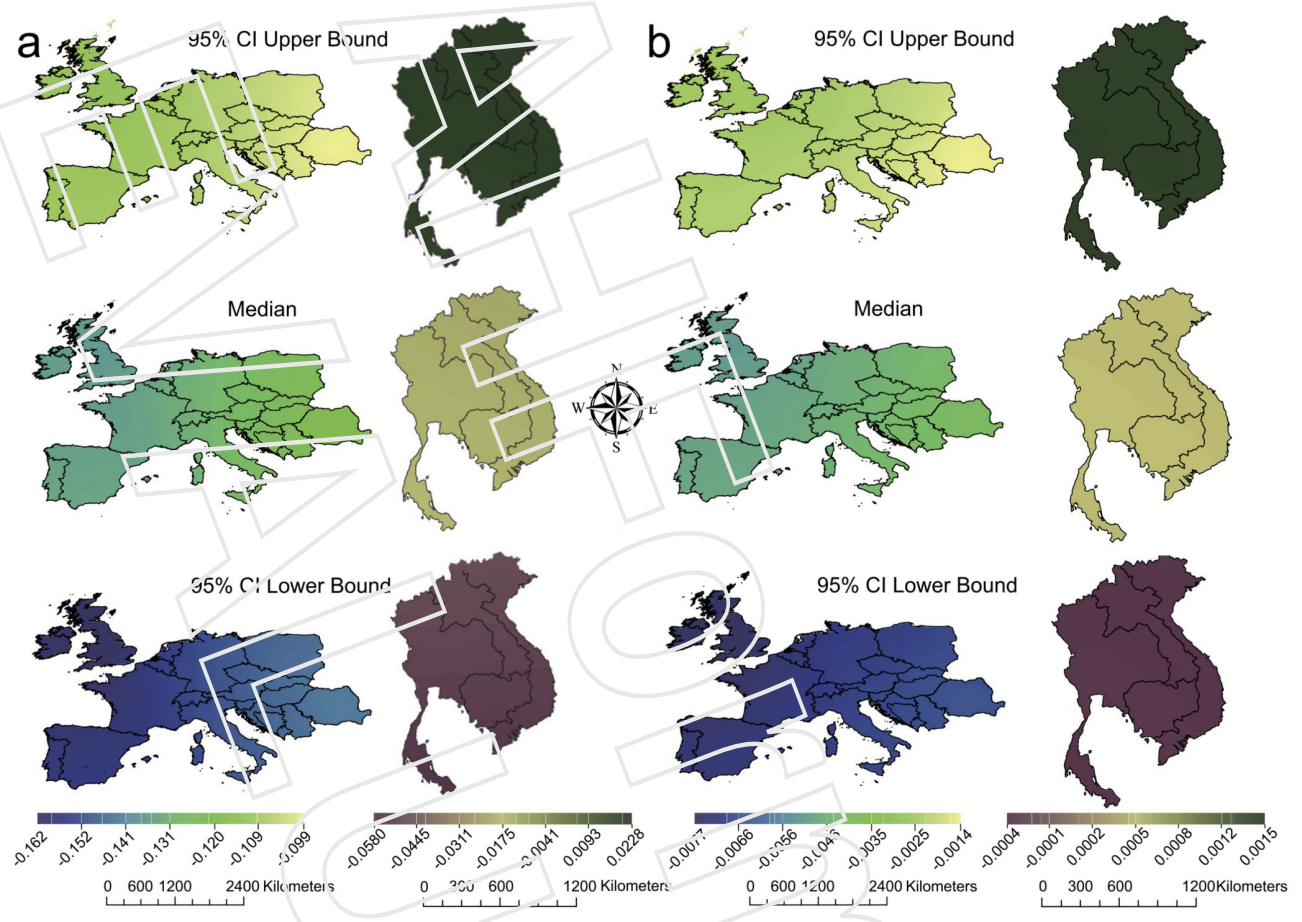
Median (middle panel), lower (bottom panel) and upper (top panel) 95% credible intervals for *φ* for European (left column) and East Asian (right column) countries. Panel (a) shows estimates for *φ*_1_ for temperature (a); panel (b) shows estimates for *φ*_2_ for precipitation. The color and map scales are listed at the bottom.

We applied the model in all 20 countries independently, using the whole dataset and with bandwidth *η* = 3000. We ran 20 independent MCMC chains for each country, and retained 10^4^ samples after discarding the first 3 × 10^3^ samples as burn-in. The pooled samples drawn from the Bayesian GWR posterior for *φ* for temperature and precipitation were used to estimate the median, lower and upper 95% bound of credible interval (CI) for each country. Figure 9 shows the results, after applying kriging interpolation with ArcGIS Version 10.7. These estimates imply that in European countries a negative association exists between influenza and both temperature and precipitation. That is, influenza transmission tends to be more prevalent during the cold and dry season. In contrast, there is no significant association in the south-east Asian countries. These conclusions are consistent with previous findings (e.g., Tamerius et al., 2013).

## 7 Conclusions

We have introduced and extended the SMI model and the candidate distribution selection technique to the field of geographic information science (GIS). Currently, a Bayesian approach for GWR models is only available for the Gaussian linear regression (Subedi et al., 2018; Ma et al., 2020). We therefore elucidate the theoretical validity of applying a Bayesian approach to generalized GWR models and reveal the essential link between the Bayesian GWR model and cutting or manipulating feedback. The motivation of Bayesian GWR model is to decrease the random error at the expense of introducing systematic error. This is realized by incorporating observations from neighbouring locations. The geographically weighted kernel manipulates the information provided by extra observations. The optimal geographical bandwidth *η* balances the trade-off between two types of error. Our model can also be applied for the Gaussian distribution with *θ* being the standard deviation. We note that our Bayesian GWR for Gaussian is different to the Bayesian GWR proposed by Ma et al. (2020). This is because our model is based on the weighted log-likelihood while Ma et al. (2020) is based on a weighted least squares approach. Specifically for the Gaussian distribution, these two models may be equivalent if the parameter of interest is only *φ* because only the exponential term of the likelihood, which is proportional to the residual sum of squares when log-likelihood is used, contains *φ*. However, they are different if *θ* is also considered.

GWR models in a frequentist framework require tedious mathematical derivation of the estimator to obtain estimates of the uncertainty of the parameter estimates, which may not be always accessible. In contrast, the Bayesian GWR model provides easily obtainable and straightforward measures of the uncertainty of the parameter estimates given the posterior samples. Furthermore, the Bayesian nature of this model means that prior knowledge can be easily introduced into the model. Unlike the SVC generalized linear model, which may require Monte Carlo sampling in a high-dimensional parameter space, the Bayesian GWR model requires only sampling separately for each location, meaning the dimension of the parameter does not scale with the number of locations, regardless of the generalized linear model used. Regarding computation, unlike the SVC model and other standard Bayesian spatial methods which require sequential sampling of parameters for all locations, the Bayesian GWR model can easily benefit from the availability of parallelization due to the separate inference for each location.

While most GWR models have considered spatially smooth parameters (coefficients), the GWR literature has not previously considered the more general case when some of the parameters are locally unique but not spatially smooth e.g. linear regression, negative binomial regression or beta regression involving a parameter akin to *θ_i_* in (1.1). Hence, our model can be viewed as an extension of the conventional GWR models that is able to simultaneously deal with (1) spatially smooth and (2) locally unique but not spatially smooth parameters. The case when all parameters vary spatially-smoothly can be incorporated into our framework by including all parameters in the *φ* vector. However, it is important to note that when *θ_i_* = ∅, the model no longer falls into the standard SMI framework.

The SMI model was previously only established for the two module case, i.e. with a single cut. In this study, we extend it to a special case of multiple cuts when information from suspect modules are manipulated via a deterministic functional form controlled by a single kernel bandwidth.

Several limitations of the current model are left for future investigation. First, the current model selects the optimal bandwidth using cross-validation. This can be computationally expensive since it requires multiple partitions of the set of observations *Y*_*i*,1:*m*_ for each location *i*. Second, although the current model can infer the parameter *θ*, this inference may suffer from insufficient observations when *m* is small because the inference of *θ* only depends on observations from the location of interest as shown in (3.3). Third, our model uses a globally fixed geographical bandwidth. This could be problematic when the true data generating process varies considerably within some areas but only varies to a small degree within other areas; or when some elements of the regression coefficient *φ* have a large geographical variation whereas other elements of *φ* have a small geographical variation. Spatially-varying bandwidth or parameter-specific distance metrics have been proposed for standard GWR models (Leong and Yue, 2017; Fotheringham et al., 2017; Lu et al., 2017; Hu et al., 2021), but the extension of these methods within a Bayesian framework is not straightforward computationally because a basic implementation would involve repeated evaluation of the geographically weighted kernel for all locations. Fourth, it would be appropriate in some applications to account for temporal effects, rather than ignoring potential temporal non-stationarity as we do in the current framework. However, while the idea is intuitive, the extension of the methodology is not straightforward because the scale of geography and time are different. This leads to a more involved bandwidth selection process, the translation of which to the Bayesian setting is not immediate, especially given our use of cross-validation and MCMC.

## Supplementary Material

Supplementary Material for “Generalized Geographically Weighted Regression Model within a Modularized Bayesian Framework” (DOI: 10.1214/22-BA1357SUPP;.pdf).

Supplementary File
