# Supplementary material for: Generalized Geographically Weighted Regression Model within a Modularized Bayesian Framework
Source: Bayesian Anal. Author manuscript; Available in PMC 2023 Jan 26. (PMC7614111; doi:10.1214/22-BA1357)
Supplement: Supplementary File [file EMS160152-supplement-Supplementary_File.pdf]

Generalized Geographically Weighted Regression Model within a Modularized Bayesian Framework

Yang Liu and Robert J.B. Goudie *

MRC Biostatistics Unit, University of Cambridge, Cambridge, UK

December 14, 2022

Supplementary Material for "Generalized Geographically Weighted Regression Model within a Modularized Bayesian Framework"

Submitted to *Bayesian Analysis*

Appendices

A Estimation error of Bayesian GWR model

To further explore the estimation error of the Bayesian GWR model, given the simulation in the main text (Section 5), we look at the estimation of individual location $\hat{\varphi}(u, v)$ to the true value $\varphi(u, v)$. An ideal model should give estimation which achieves $\hat{\varphi} = \varphi$. However, estimation will inevitably affected by error. Notice that, although we have run a Bayesian

*E-mail address: yang.liu@mrc-bsu.cam.ac.uk (Yang Liu)

GWR model independently at each of the 1600 locations, they share the same geographical bandwidth η. Therefore, it is reasonable to assume that the degree of error introduced due to the model should be similar for all locations. Here, we assume the following equation:

$$\hat{\varphi}(u,v) = F(\varphi(u,v)) + \varepsilon(u,v),$$

where F is a unknown deterministic function and $\varepsilon(u,v)$ *i.i.d* follows an arbitrary distribution with mean 0 and variance σ^2. The term $\varepsilon(u,v)$ describes the random error that naturally arises from samples due to the randomness of observations and can be reduced by increasing the sample size. The squared error is $(F(\varphi(u,v)) + \varepsilon(u,v) - \varphi(u,v))^2$. The function F describes the systematic error that is due to the misspecification (i.e., the use of samples from neighbouring locations). When a coefficient varies geographically, this systematic error can not be removed if we include neighbouring samples. If there is no systematic error (i.e., we do not borrow any sample from neighbouring locations or coefficient does not vary across the space), then $F(\varphi(u,v)) = \varphi(u,v)$.

Figure A1 shows a scatter plot of the estimated mean coefficients at each of the geographic locations for each bandwidth choice, against their true values. The estimates when $\eta = 0.0001$ distribute evenly around the true values because this model emphasizes local characteristics, but with large variance due to the large random error due to the insufficient number of samples used by this model. In contrast, the estimates when $\eta = 1000$ are relatively horizontal because this model assumes coefficients are relatively constant across the geographical space, leading to a large deviation from the true values due to the systematic error caused by including too much information from neighbouring geographic locations. The model with the optimal bandwidth $\eta = 4$ has less systematic error than $\eta = 1000$, and much smaller random error than $\eta = 0.0001$.

Given the clear linear trend for all bandwidth choices, we assume a linear form $F(\varphi(u,v)) = a + b\varphi(u,v)$ for the systematic error, and summarise the results via the linear regression coefficients (Table A1). We first consider the systematic error. When $\eta = 0.0001$ and $\eta = 4$, the intercept is close to 0 and the slope is close to 1, indicating the systematic error is very small. In contrast when $\eta = 1000$, the intercept differs from 0 and the slope is clearly not close to 1. This confirms the systematic error we discussed before. Specifically, the slope goes to 0 when $\eta = 1000$. This again reveals the fact that larger geographical bandwidth ignores geographical variation. Now we consider the random error. It is clear that, for both φ_1 and φ_2, the model with $\eta = 1000$ gives the smallest random error σ and σ increases as η decreases. This trend reveals the varying pattern of the dispersion thanks to the changing of the sample size. In summary, the model with $\eta = 4$ balances both systematic and random error.

Coefficient	Bandwidth	$\hat{a}$	$\hat{b}$	$\hat{\sigma}$
	0.0001	-0.0428	1.1041	0.0249
φ_1	4	-0.0445	1.1169	0.0236
	1000	0.4775	0.0003	0.0003
	0.0001	0.0597	0.6857	0.0492
φ_2	4	0.0901	0.5395	0.0061
	1000	0.2213	0.0001	0.0003

Table A1: Estimated intercept $\hat{a}$, slope $\hat{b}$ and standard deviation $\hat{\sigma}$ under the linear model of the estimated mean coefficients for each choice of geographic bandwidth.

B The setting of PICAR model

Let s_i, $i = 1, \cdots, n$, denote the index of n locations. X_1, X_2 and X_3 are the first, second and third columns of X. A is the projector matrix and M is the Moran's basis function matrix. δ denotes basis coefficients for the spatially varying coefficients. Q is the precision matrix for the latent spatial field located on the mesh vertices and τ denotes the precision parameters. $\otimes$ represents the Hadamard product. Mesh is generated on the 40×40 regular lattice by using INLA package and the number of Moran's basis function is 85 which yields the lowest out-of-sample cross-validated mean squared prediction error. The PICAR model that we used in the simulation section is:

1. Data model:

$$
\begin{aligned}
&Y(s_i)|Pr(s_i) \sim \mathbf{NegBin}(\text{Probability} = Pr(s_i), \text{size} = 1/0.5),\\
&Pr(s_i) = \frac{1}{1 + 0.5\exp(\eta(s_i))},\\
&\eta = (\eta(s_1), \cdots, \eta(s_n)),\\
&\eta = X\varphi + X_1 \otimes AM\delta_1 + X_2 \otimes AM\delta_2 + X_3 \otimes AM\delta_3
\end{aligned}
$$

2. Process model:

$$
\begin{aligned}
&\delta_1 \sim \mathbf{N}(0, \tau_1^{-1}(M'QM)^{-1}),\\
&\delta_2 \sim \mathbf{N}(0, \tau_2^{-1}(M'QM)^{-1}),\\
&\delta_3 \sim \mathbf{N}(0, \tau_3^{-1}(M'QM)^{-1})
\end{aligned}
$$

3. Parameter model:

$$\begin{aligned}
\tau_1 &\sim \mathbf{Gamma}(0.5, 1/2000), \\
\tau_2 &\sim \mathbf{Gamma}(0.5, 1/2000), \\
\tau_3 &\sim \mathbf{Gamma}(0.5, 1/2000), \\
\varphi_1 &\sim \mathbf{N}(0, 100), \\
\varphi_2 &\sim \mathbf{N}(0, 100), \\
\varphi_3 &\sim \mathbf{N}(0, 100)
\end{aligned}$$

C Additional Simulations with Different Number of Observations

In this section we consider the cases when the number of replicates at each location $m = 10$ and $m = 50$. All the other settings of the model, MCMC algorithm and computational environment are the same as the original simulation example.

We first look at the difference between the optimal bandwidth under different numbers of replicates. Figures A2 and A3 show the elpd against geographical bandwidth when number of replicates are $m = 50$ and $m = 10$ (both considerably less than the original $m = 100$ in the main text). From these figures, we can conclude that the optimal geographical bandwidth is getting larger as the number of replicates at each location is decreasing (bandwidth 8 for $m = 50$ replicates case and bandwidth 10 for $m = 10$ replicates case). This is consistent with our expectation: when the number of replicates decreases, the information from a single location decreases, and hence, in order to achieve reliable estimation, the method selects a larger bandwidth so that more data can be included into the analysis. In addition, we notice that the range of elpd when the bandwidth is only 0.0001 under 10 replicates case is very large. This raises the concern of potentially unreliable cross-validation due to an insufficient number of observations (only 5 observations are used to estimate the coefficient).

After setting the optimal bandwidth, we apply our Bayesian GWR methodology to obtain the estimated geographically varying coefficients shown in Figure A4 and A5 and the corresponding MSE shown in Figure A6 and A7. It is clear that our model with the corresponding optimal bandwidths ($\eta = 8$ with $m = 50$ replicates; and $\eta = 10$ with $m = 10$ replicates) still gives a reasonable and better estimation.

D Proof of the Lemma 3.1

Proof. We prove the lemma when $n = 2$; the proof can be easily extended to case when $n > 2$ by induction.

Given the posterior $p(\theta_{0:n}, \varphi_0|Y_{0:n,1:m})$, we have

$$p(\theta_0, \theta_1, \theta_2, \varphi_0|Y_{0:2,1:m}) = p(\theta_2|\theta_0, \theta_1, \varphi_0, Y_{0:2,1:m})p(\theta_0, \theta_1, \varphi_0|Y_{0:2,1:m}).$$

Then by conditional independence of θ_2 and $(Y_{0:1,1:m}, \theta_{0:1})$ given φ_0, we have

$$p(\theta_0, \theta_1, \theta_2, \varphi_0|Y_{0:2,1:m}) = p(\theta_2|Y_{2,1:m}, \varphi_0)p(\theta_0, \theta_1, \varphi_0|Y_{0:2,1:m}).$$

For the term $p(\theta_0, \theta_1, \varphi_0|Y_{0:2,1:m})$, we have

$$\begin{aligned} p(\theta_0, \theta_1, \varphi_0|Y_{0:2,1:m}) &= p(\theta_1|\theta_0, \varphi_0, Y_{0:2,1:m})p(\theta_0, \varphi_0|Y_{0:2,1:m}) \\ &= \int p(\theta_1|\theta_0, \theta_2, \varphi_0, Y_{0:2,1:m})p(\theta_2|\theta_0, \varphi_0, Y_{0:2,1:m})d\theta_2 \; p(\theta_0, \varphi_0|Y_{0:2,1:m}). \end{aligned}$$

Similarly, by conditional independence of θ_1 and $(Y_{-1,1:m}, \theta_{-1})$ given φ

$$\begin{aligned} p(\theta_0, \theta_1, \varphi_0|Y_{0:2,1:m}) &= \int p(\theta_1|Y_{1,1:m}, \varphi_0)p(\theta_2|\theta_0, \varphi_0, Y_{0:2,1:m})d\theta_2 \; p(\theta_0, \varphi_0|Y_{0:2,1:m}) \\ &= p(\theta_1|Y_{1,1:m}, \varphi_0)p(\theta_0, \varphi_0|Y_{0:2,1:m}). \end{aligned}$$

Hence, we have:

$$p(\theta_0, \theta_1, \theta_2, \varphi_0|Y_{0:2,1:m}) = p(\theta_2|Y_{2,1:m}, \varphi_0)p(\theta_1|Y_{1,1:m}, \varphi_0)p(\theta_0, \varphi_0|Y_{0:2,1:m}).$$

□

E Proof of the Theorem 3.1

Proof. We first notice that the following equality holds:

$$\begin{aligned} &\mathop{\mathbb{E}}_{Y_{0:n,1:m} \sim \check{P}_{0:n,1:m}} \exp\left(-r_{0,1:m}(\psi) - \sum_{i=1}^{n} W_i r_{i,1:m}(\psi)\right) \\ &= \int \frac{p(Y_{0,1:m}|\theta_0, \varphi_0)}{\check{p}_{0,1:m}(Y_{0,1:m})} \prod_{i=1}^{n} \left(\frac{p(Y_{i,1:m}|\tilde{\theta}_i, \varphi_0)}{\check{p}_{i,1:m}(Y_{i,1:m})}\right)^{W_i} \check{P}_{0:n,1:m}(dY_{0:n,1:m}) \\ &= \prod_{i=1}^{n} \int \left(\frac{p(Y_{i,1:m}|\tilde{\theta}_i, \varphi_0)}{\check{p}_{i,1:m}(Y_{i,1:m})}\right)^{W_i} \check{P}_{i,1:m}(dY_{i,1:m}) \end{aligned}$$

For an arbitrary single term, it is straightforward to have that

$$\begin{aligned}
&\int \left(\frac{p(Y_{i,1:m}|\tilde{\theta}_i, \varphi_0)}{\check{p}_{i,1:m}(Y_{i,1:m})} \right)^{W_i} \check{P}_{i,1:m}(dY_{i,1:m}) \\
&= \exp\left(\log\left\{ \int (p(Y_{i,1:m}|\tilde{\theta}_i, \varphi_0))^{W_i} (\check{p}_{i,1:m}(Y_{i,1:m}))^{1-W_i} dY_{i,1:m} \right\} \right) \\
&= \exp\left(-m(1-W_i)\mathbb{D}_{W_i}\left(p(\cdot|\tilde{\theta}_i, \varphi_0), \check{p}_i(\cdot) \right) \right).
\end{aligned}$$

Hence, it follows that

$$\begin{aligned}
&\mathop{\mathbb{E}}_{Y_{0:n,1:m}\sim \check{P}_{0:n,1:m}} \exp\left(-r_{0,1:m}(\psi) - \sum_{i=1}^{n} W_i r_{i,1:m}(\psi) \right) \\
&= \exp\left(-\sum_{i=1}^{n} m(1-W_i)\mathbb{D}_{W_i}\left(p(\cdot|\tilde{\theta}_i, \varphi_0), \check{p}_i(\cdot) \right) \right).
\end{aligned}$$

Now move the right hand side term to the left side and multiply ε, so we obtain

$$\begin{aligned}
&\mathop{\mathbb{E}}_{Y_{0:n,1:m}\sim \check{P}_{0:n,1:m}} \exp\left\{ -r_{0,1:m}(\psi) - \sum_{i=1}^{n} W_i r_{i,1:m}(\psi) \right. \\
&\quad \left. + \sum_{i=1}^{n} m(1-W_i)\mathbb{D}_{W_i}\left(p(\cdot|\tilde{\theta}_i, \varphi_0), \check{p}_i(\cdot) \right) - \log(\frac{1}{\varepsilon}) \right\} \\
&= \varepsilon.
\end{aligned}$$

Now we calculate the expectation with respect to prior Π and exchange expectations by Fubini's theorem.

$$\begin{aligned}
&\mathop{\mathbb{E}}_{Y_{0:n,1:m}\sim \check{P}_{0:n,1:m}} \mathop{\mathbb{E}}_{\psi\sim\Pi} \exp\left\{ -r_{0,1:m}(\psi) - \sum_{i=1}^{n} W_i r_{i,1:m}(\psi) \right. \\
&\quad \left. + \sum_{i=1}^{n} m(1-W_i)\mathbb{D}_{W_i}\left(p(\cdot|\tilde{\theta}_i, \varphi_0), \check{p}_i(\cdot) \right) - \log(\frac{1}{\varepsilon}) \right\} \\
&= \varepsilon.
\end{aligned}$$

The Donsker-Varadhan's change of measure states that for any measurable function $\Upsilon : \Psi \to \mathbb{R}$, we have

$$\mathop{\mathbb{E}}_{\psi\sim F} \Upsilon(\psi) \le \mathbb{D}_{KL}(f(\cdot), \pi(\cdot)) + \log\left(\mathop{\mathbb{E}}_{\psi\sim\Pi} \exp(\Upsilon(\psi)) \right).$$

By applying this Donsker-Varadhan's change of measure on the left side of the above equality, we have

$$\begin{aligned}&\mathop{\mathbb{E}}_{Y_{0:n,1:m}\sim\check{P}_{0:n,1:m}}\exp\Bigg\{\mathop{\mathbb{E}}_{\psi\sim F}\Bigg(-r_{0,1:m}(\psi)-\sum_{i=1}^{n}W_i r_{i,1:m}(\psi)\\&\quad+\sum_{i=1}^{n}m(1-W_i)\mathbb{D}_{W_i}\left(p(\cdot|\tilde{\theta}_i,\varphi_0),\check{p}_i(\cdot)\right)\Bigg)-\log(\frac{1}{\varepsilon})-\mathbb{D}_{KL}(f(\cdot),\pi(\cdot))\Bigg\}\\&\le\varepsilon.\end{aligned}$$

By applying the Markov's inequality, with $\check{P}_{0:n,1:m}$ probability at least $(1-\varepsilon)$, we have

$$\begin{aligned}&\exp\Bigg\{\mathop{\mathbb{E}}_{\psi\sim F}\Bigg(-r_{0,1:m}(\psi)-\sum_{i=1}^{n}W_i r_{i,1:m}(\psi)\\&\quad+\sum_{i=1}^{n}m(1-W_i)\mathbb{D}_{W_i}\left(p(\cdot|\tilde{\theta}_i,\varphi_0),\check{p}_i(\cdot)\right)\Bigg)-\log(\frac{1}{\varepsilon})-\mathbb{D}_{KL}(f(\cdot),\pi(\cdot))\Bigg\}\\&\le 1.\end{aligned}$$

Remove the exponential function and multiply $1/m$, we have the following inequality holds

$$\begin{aligned}&\int\sum_{i=1}^{n}(1-W_i)\mathbb{D}_{W_i}\left(p(\cdot|\tilde{\theta}_i,\varphi_0),\check{p}_i(\cdot)\right)F(d\psi)\\&\quad\le\frac{1}{m}\int\left(r_{0,1:m}+\sum_{i=1}^{n}W_i r_{i,1:m}\right)F(d\psi)+\frac{\mathbb{D}_{KL}(f(\cdot),\pi(\cdot))}{m}+\frac{1}{m}\log\left(\frac{1}{\varepsilon}\right)\end{aligned}$$

with $\check{P}_{0:n,1:m}$ probability at least $(1-\varepsilon)$. □

F Proof of the remark of Theorem 3.1

Proof. Given the inequality in Theorem 3.1, the left hand side of the inequality can be modified as

$$\begin{aligned}
&\frac{1}{m}\mathop{\mathbb{E}}_{\psi\sim F}\left\{-\sum_{i=1}^{n}\log\left(\mathop{\mathbb{E}}_{Y_{i,1:m}\sim\check{P}_{i,1:m}}\left(\frac{p(Y_{i,1:m}|\tilde{\theta}_i,\varphi_0)}{\check{p}_{i,1:m}(Y_{i,1:m})}\right)^{W_i}\right)\right\}\\
&=\mathop{\mathbb{E}}_{\psi\sim F}\left\{-\log\left(\mathop{\mathbb{E}}_{Y_{0:n,1:m}\sim\check{P}_{0:n,1:m}}\exp\left(-mL_{1:m}(\psi)\right)\right)^{\frac{1}{m}}\right\}\\
&\geq\mathop{\mathbb{E}}_{\psi\sim F}\left\{-\log\left(\mathop{\mathbb{E}}_{Y_{0:n,1:m}\sim\check{P}_{0:n,1:m}}\exp\left(-L_{1:m}(\psi)\right)\right)\right\},
\end{aligned}$$

and the right hand side of the inequality can be rewritten as:

$$\mathop{\mathbb{E}}_{\psi\sim F}L_{1:m}(\psi)+\frac{\mathbb{D}_{KL}(f(\cdot),\pi(\cdot))}{m}+\frac{1}{m}\log\left(\frac{1}{\varepsilon}\right).$$

Hence, we have derived the "information posterior bound". □

G Proof of the Theorem 3.2

Proof. We rewrite the criterion function as:

$$M_m(f(\psi))=\mathbb{D}_{KL}(f(\cdot),\pi(\cdot))-\int f(\psi)\log\left(p_{\text{pow}}(Y_{0:n,1:m}|\psi)\right)d\psi.$$

Minimizing $M_m(f(\psi))$ is equivalent to minimizing:

$$\begin{aligned}
\Delta I(f(\psi))&=\int f(\psi)\log(f(\psi))d\psi-\int f(\psi)\log(\pi(\psi))d\psi+\log(p_{\text{pow}}(Y_{0:n,1:m}))\\
&\quad-\int f(\psi)\log\left(p_{\text{pow}}(Y_{0:n,1:m}|\psi)\right)d\psi\\
&=\int f(\psi)\log\left(\frac{f(\psi)p_{\text{pow}}(Y_{0:n,1:m})}{p_{\text{pow}}(Y_{0:n,1:m}|\psi)\pi(\psi)}\right)d\psi\\
&=\int f(\psi)\log\left(\frac{f(\psi)}{p_{\text{pow}}(\psi|Y_{0:n,1:m})}\right)d\psi
\end{aligned}$$

Obviously we have:

$$\int p_{\text{pow}}(\psi|Y_{0:n,1:m})d\psi = \int \frac{p_{\text{pow}}(Y_{0:n,1:m}|\psi)\pi(\psi)}{p_{\text{pow}}(Y_{0:n,1:m})}d\psi = 1,$$

so geographically-powered posterior $P_{\text{pow}}(\psi|Y_{0:n,1:m})$ is a proper probability distribution and therefore we can write $\Delta I(f(\psi))$ as an Kullback-Leibler divergence:

$$\Delta I(f(\psi)) = \mathbb{D}_{KL}\left(f(\cdot), \frac{p_{\text{pow}}(Y_{0:n,1:m}|\cdot)\pi(\cdot)}{p_{\text{pow}}(Y_{0:n,1:m})}\right) \geq 0.$$

It is clear that $f(\psi) = p_{\text{pow}}(\psi|Y_{0:n,1:m})$ minimizes the criterion function $M_m(f(\psi))$ by reducing the difference of input and output information $\Delta I(f(\psi))$ to 0 and thus it results from an optimal information processing rule. □

H Proof of the Theorem 3.3

Proof. Note that, this theorem easily follows the result of Theorem 3.1. Here we provide a different way to prove it.

According to Theorem 3.2, $f(\psi) = p_{\text{pow}}(\psi|Y_{0:n,1:m})$ minimizes $M_m(f(\psi))$. Meanwhile, minimizing $M_m(f(\psi))$ is equivalent to minimizing:

$$\begin{aligned}
K_m(f(\psi)) = m^{-1} \int f(\psi) \log\left(\check{p}_{0:n,1:m}(Y_{0:n,1:m})\right) d\psi + m^{-1}\mathbb{D}_{KL}(f(\cdot), \pi(\cdot)) \\
- m^{-1} \int f(\psi) \log\left(p(Y_{0,1:m}|\theta_0, \varphi_0)\right) d\psi \\
- m^{-1} \sum_{i=1}^{n} W_i \int f(\psi) \log\left(p(Y_{i,1:m}|\tilde{\theta}_i, \varphi_0)\right) d\psi \\
= m^{-1} \int f(\psi) \log\left(\frac{\check{p}_{0:n,1:m}(Y_{0:n,1:m})}{P_{\text{pow}}(Y_{0:n,1:m}|\psi)}\right) d\psi \\
+ m^{-1}\mathbb{D}_{KL}(f(\cdot), \pi(\cdot))
\end{aligned}$$

Denote the jth batch of observations from all locations by $Y_{0:n,j} = (Y_{0,j}, Y_{1,j}, ..., Y_{n,j})$. By

independence:

$$
\begin{aligned}
K_m(f(\psi)) &= m^{-1}\int f(\psi)\sum_{j=1}^{m}\log\left(\frac{\check{p}(Y_{0:n,j})}{p(Y_{0,j}|\theta_0,\varphi_0)\prod_{i=1}^{n}p(Y_{i,j}|\tilde{\theta}_i,\varphi_0)^{W_i}}\right)d\psi\\
&\quad + m^{-1}\mathbb{D}_{KL}(f(\cdot),\pi(\cdot))\\
&= m^{-1}\int f(\psi)\sum_{j=1}^{m}\left\{\log\left(\frac{\check{p}_0(Y_{i,j})}{p(Y_{i,j}|\theta_0,\varphi_0)}\right)+\sum_{i=1}^{n}W_i\log\left(\frac{\check{p}_i(Y_{i,j})}{p(Y_{i,j}|\tilde{\theta}_i,\varphi_0)}\right)\right\}d\psi\\
&\quad + \Lambda^{(m)} + m^{-1}\mathbb{D}_{KL}(f(\cdot),\pi(\cdot)),\\
&= \Lambda^{(m)} + \mathop{\mathbb{E}}_{\psi\sim F}L_{1:m}(\psi) + m^{-1}\mathbb{D}_{KL}(f(\cdot),\pi(\cdot)),
\end{aligned}
$$

where

$$
\Lambda^{(m)} := m^{-1}\sum_{j=1}^{m}\left(\sum_{i=1}^{n}(1-W_i)\log(\check{p}_i(Y_{i,j})\right).
$$

is a constant. Hence we have the geographically-powered posterior P_{pow} minimizes

$$
\mathop{\mathbb{E}}_{\psi\sim P_{\text{pow}}}L_{1:m}(\psi) + m^{-1}\mathbb{D}_{KL}(p_{\text{pow}}(\cdot|Y_{0:n,1:m}),\pi(\cdot)).
$$

When $m\to\infty$, we have $K_m(f(\psi))$ converges to:

$$
\begin{aligned}
K_\infty(f(\psi)) &= \int f(\psi)\int \check{p}_{0:n,1:m}(Y_{0:n,1:m})\log\left(\frac{\check{p}_{0:n,1:m}(Y_{0:n,1:m})}{p(y_0|\theta_0,\varphi_0)\prod_{i=1}^{n}p(y_i|\tilde{\theta}_i,\varphi_0)^{W_i}}\right)dY_{0:n,1:m}d\psi\\
&= \int f(\psi)\int \check{p}_{0:n,1:m}(Y_{0:n,1:m})\log\left(\frac{\check{p}_0(y_0)}{p(y_0|\theta_0,\varphi_0)}\right)dY_{0:n,1:m}d\psi\\
&\quad + \int f(\psi)\int \check{p}_{0:n,1:m}(Y_{0:n,1:m})\sum_{i=1}^{n}\log\left(\frac{\check{p}_i(y_i)}{p(y_i|\tilde{\theta}_i,\varphi_0)^{W_i}}\right)dY_{0:n,1:m}d\psi.
\end{aligned}
$$

We now look at an arbitrary single term and decompose it:

$$\begin{aligned}
&\int f(\psi) \int \check{p}_{0:n,1:m}(Y_{0:n,1:m}) \log\left(\frac{\check{p}_i(y_i)}{p(y_i|\tilde{\theta}_i,\varphi_0)^{W_i}}\right) dY_{0:n,1:m} d\psi \\
&= \int f(\psi)(1-W_i) \int \check{p}_i(y_i)\log(\check{p}_i(y_i)) dy_i d\psi \\
&\quad + \int f(\psi) W_i \int \check{p}_i(y_i) \log\left(\frac{\check{p}_i(y_i)}{p(y_i|\tilde{\theta}_i,\varphi_0)}\right) dy_i d\psi \\
&= (1-W_i)\int \check{p}_i(y_i)\log(\check{p}_i(y_i)) dy_i + W_i \int f(\psi) \mathbb{D}_{KL}(\check{p}_i(\cdot), p(\cdot|\tilde{\theta}_i,\varphi_0)) d\psi \\
&= (1-W_i)\int \check{p}_i(y_i)\log(\check{p}_i(y_i)) dy_i + W_i \mathop{\mathbb{E}}_{\psi\sim P_{\text{pow}}^{(\infty)}} \left(\mathbb{D}_{KL}(\check{p}_i(\cdot), p(\cdot|\tilde{\theta}_i,\varphi_0))\right),
\end{aligned}$$

where the expectation is calculated with respect to distribution $f(\psi)$. We denote a constant Λ as:

$$\Lambda = \sum_{i=1}^{n}(1-W_i)\int \check{p}_i(y_i)\log(\check{p}_i(y_i))dy_i.$$

We then have

$$\begin{aligned}
K_\infty(f(\psi)) &= \Lambda + \mathop{\mathbb{E}}_{\psi\sim P_{\text{pow}}^{(\infty)}}\left(\mathbb{D}_{KL}(\check{p}_0(\cdot), p(\cdot|\theta_0,\varphi_0)) + \sum_{i=1}^{n} W_i \mathbb{D}_{KL}(\check{p}_i(\cdot), p(\cdot|\tilde{\theta}_i,\varphi_0))\right) \\
&= \Lambda + \mathop{\mathbb{E}}_{\psi\sim P_{\text{pow}}^{(\infty)}} L(\psi).
\end{aligned}$$

According to Theorem 3.2 and assuming the probability measure $P_{\text{pow}}^{(\infty)}$ exists, $f(\psi) = p_{\text{pow}}^{(\infty)}(\psi|Y_{0:n,1:\infty})$ minimizes $K_\infty(f(\psi))$. Since Λ is a constant, the geographically-powered posterior $p_{\text{pow}}^{(\infty)}(\psi|Y_{0:n,1:\infty})$ is required to put all its mass at $\psi^* = (\theta_0^*, \tilde{\theta}_{1:n}^*, \varphi_0^*)$ when $m \to \infty$, where ψ^* satisfies:

$$\psi^* = \mathop{\arg\min}_{\psi=(\theta_0,\tilde{\theta}_{1:n},\varphi_0)} \mathbb{D}_{KL}\left(\check{p}_0(\cdot), p(\cdot|\theta_0,\varphi_0)\right) + \sum_{i=1}^{n} W_i \mathbb{D}_{KL}\left(\check{p}_i(\cdot), p(\cdot|\tilde{\theta}_i,\varphi_0)\right). \qquad \square$$

I Proof of the Theorem 4.1

Proof. To obtain the best predictive performance for new observations $Y^*_{0:n}$ from locations (u_i, v_i), $i = 0, ..., n$, we need to maximise the expected log pointwise predictive density for $Y^*_{0:n}$. Let ψ_{M_i} be the corresponding parameters of model M_i, $i = 0, ..., n$. By the assumption of the geographically weighted regression model (i.e., observation Y is independently generated from the true data generating process, we have

$$p(Y^*_{0:n}|\psi_{M_0}, ..., \psi_{M_n}) = \prod_{i=0}^{n} p(Y^*_i|\psi_{M_0}, ..., \psi_{M_n}) = \prod_{i=0}^{n} p(Y^*_i|\psi_{M_i}).$$

By the Assumption 4.2, we have

$$p(\psi_{M_0}, ..., \psi_{M_n}|Y_{0:n,1:m}) = \prod_{i=0}^{n} p_{M_i}(\psi_{M_i}|Y_{0:n,1:m}).$$

Then we have

$$\begin{aligned} p(Y^*_{0:n}|Y_{0:n,1:m}) &= \int p(Y^*_{0:n}|\psi_{M_0}, ..., \psi_{M_n})p(\psi_{M_0}, ..., \psi_{M_n}|Y_{0:n,1:m})d\psi_{M_0}...d\psi_{M_n} \\ &= \prod_{i=0}^{n} \int p(Y^*_i|\psi_{M_i})p_{M_i}(\psi_{M_i}|Y_{0:n,1:m})d\psi_{M_i}. \end{aligned}$$

Plugging it into the expected log pointwise predictive density for $Y^*_{0:n}$, we have

$$\begin{aligned} \text{elpd}(M) &= \int \log\left(p(Y^*_{0:n}|Y_{0:n,1:m})\right) \prod_{i=0}^{n} \check{p}_i(Y^*_i)dY^*_{0:n} \\ &= \sum_{i=0}^{n} \int \left(\log\left(\int p(Y^*_i|\psi_{M_i})p_{M_i}(\psi_{M_i}|Y_{0:n,1:m})d\psi_{M_i} \right) \prod_{j=0}^{n} \check{p}_j(Y^*_j) \right) dY^*_{0:n} \\ &= \sum_{i=0}^{n} \int \left(\log\left(\int p(Y^*_i|\psi_{M_i})p_{M_i}(\psi_{M_i}|Y_{0:n,1:m})d\psi_{M_i} \right) \check{p}_i(Y^*_i) \right) dY^*_i \\ &= \sum_{i=0}^{n} \int \check{p}_i(Y^*_i) \log(p_{M_i}(Y^*_i|Y_{0:n,1:m}))dY^*_i \\ &= \sum_{i=0}^{n} \text{elpd}_{(u_i,v_i)}(M_i). \end{aligned}$$

Given any geographically weighted regression model $M_i = ((u_i', v_i'), \eta)$, by Assumption 4.1, we have that for $\forall \eta > 0$, $M_i(\eta) = ((u_i, v_i), \eta)$ always maximizes the elpd. That is

$$\text{elpd}_{(u_i,v_i)}(M_i) \le \text{elpd}_{(u_i,v_i)}(M_i(\eta)),\ \ i = 0, ..., n.$$

Now we have

$$\text{elpd}(M) \le \sum_{i=0}^{n} \text{elpd}_{(u_i,v_i)}(M_i(\eta)).$$

This has proved $(u_i^*, v_i^*) = (u_i, v_i)$ for all i. To further maximize $\text{elpd}(M)$, we simply require

$$\eta^* = \arg\max_{\eta} \frac{1}{n+1} \sum_{i=0}^{n} \text{elpd}_{(u_i,v_i)}(M_i(\eta)).$$

□

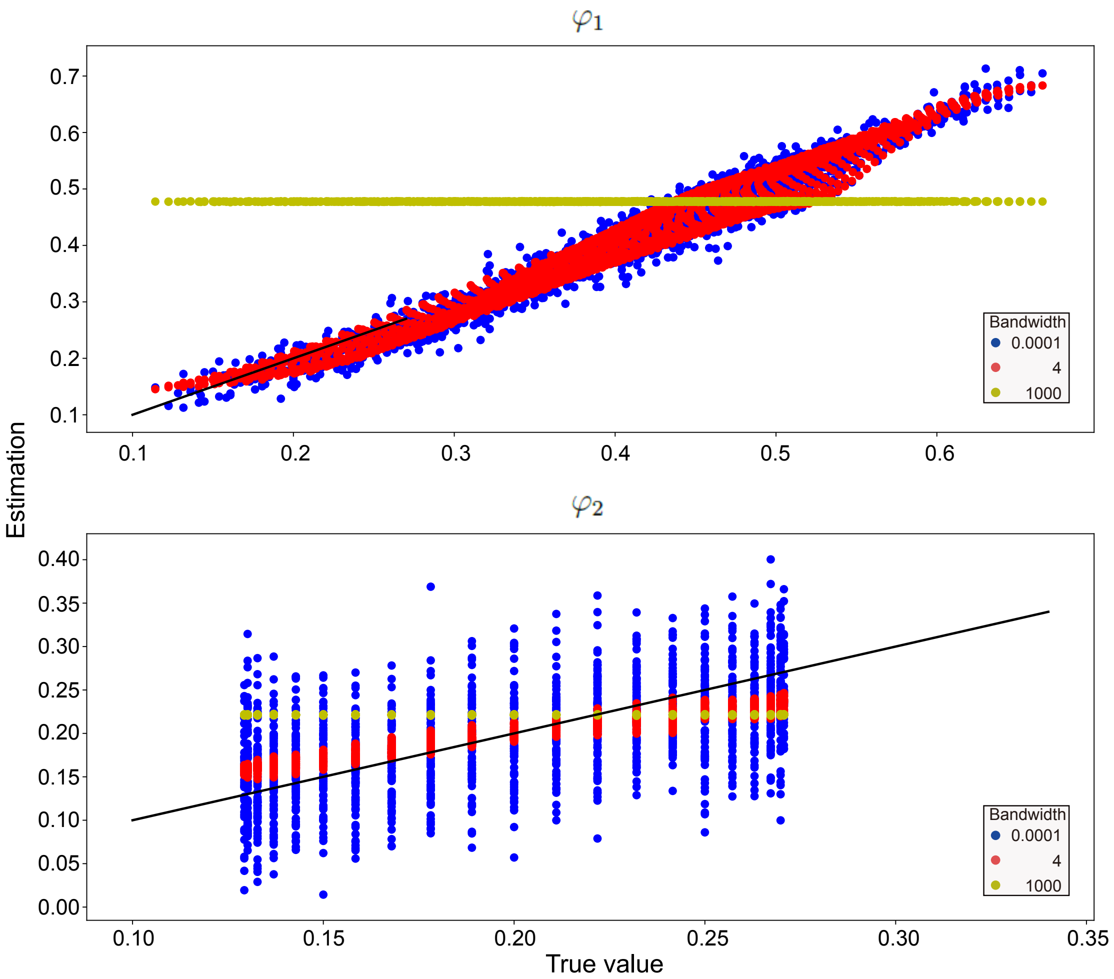

Figure A1: **Scatter plot of the estimated mean coefficient for ψ_1 and ψ_2 under the Bayesian GWR model against the true value at each of the 1600 geographic locations.** Results are shown for geographic bandwidths $\eta = 0.0001$ (blue), $\eta = 4$ (red) and $\eta = 1000$ (yellow). The diagonal benchmark line $\hat{\varphi} = \varphi$ indicates where the estimates should be centred around if there is no systematic error.

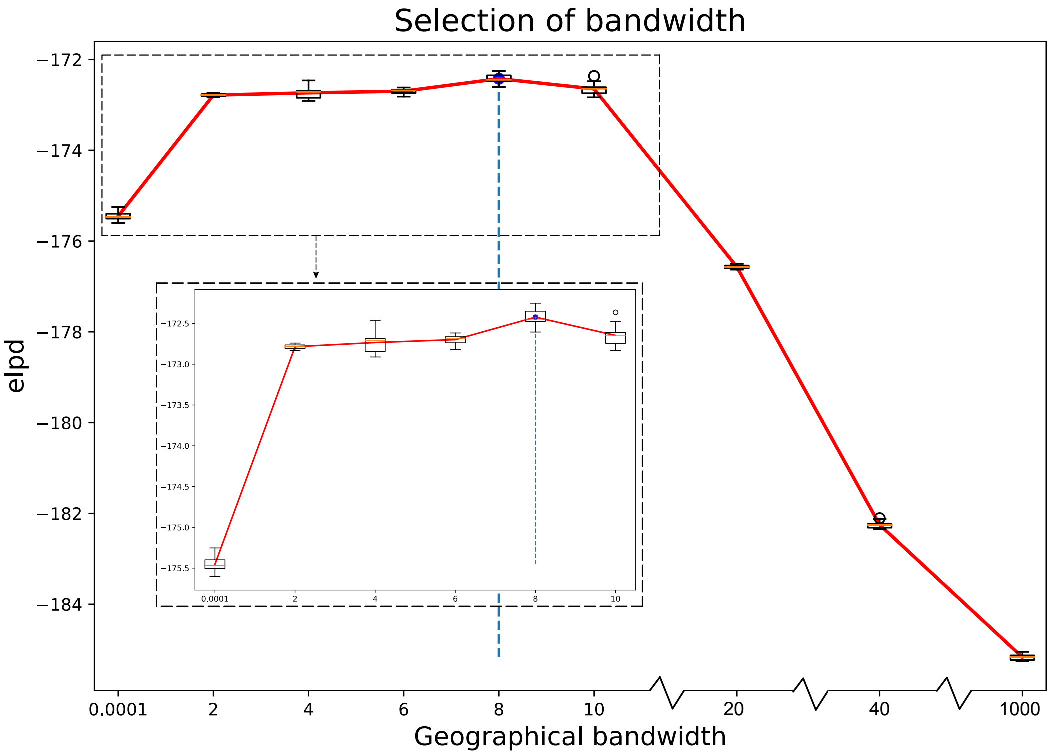

Figure A2: **elpd against geographical bandwidth when number of observations is 50.** Each boxplot represents the elpd estimates from 10 chains across the whole geographic space. The red line is the average elpd estimates across the 10 chains. The blue dashed line indicates the optimal bandwidth. Two balck dashed areas are equivalent: the inset figure is a zoomed-in version.

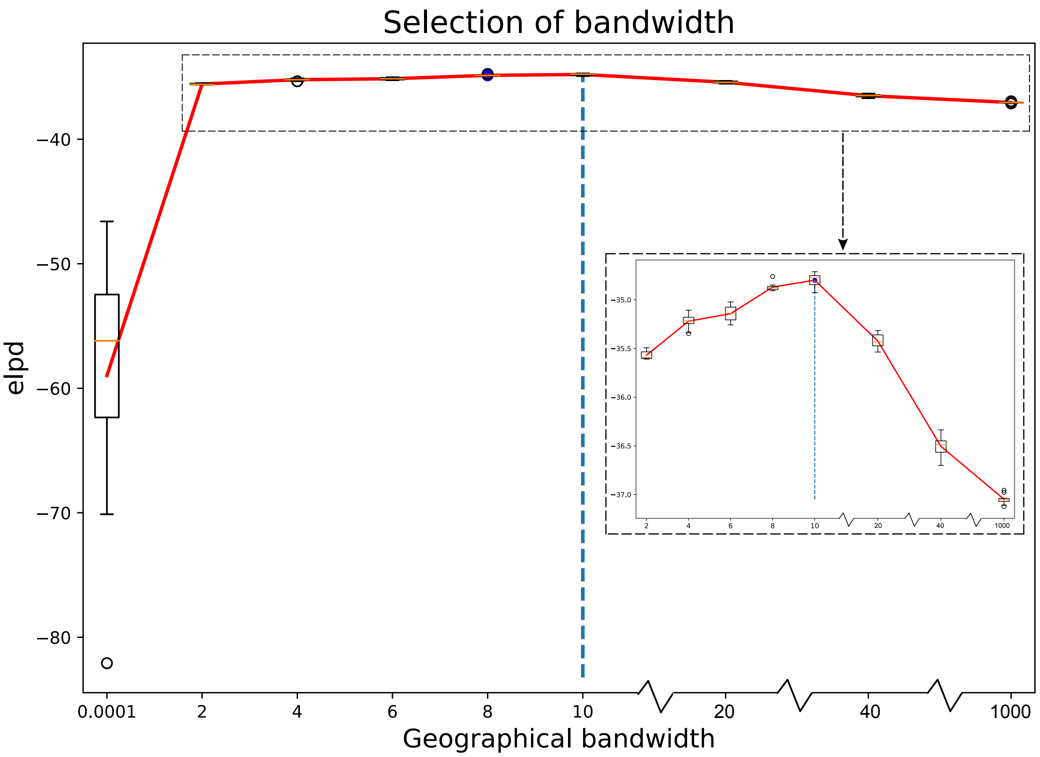

Figure A3: **elpd against geographical bandwidth when number of observations is 10.** Each boxplot represents the elpd estimates from 10 chains across the whole geographic space. The red line is the average elpd estimates across the 10 chains. The blue dashed line indicates the optimal bandwidth. Two balck dashed areas are equivalent: the inset figure is a zoomed-in version.

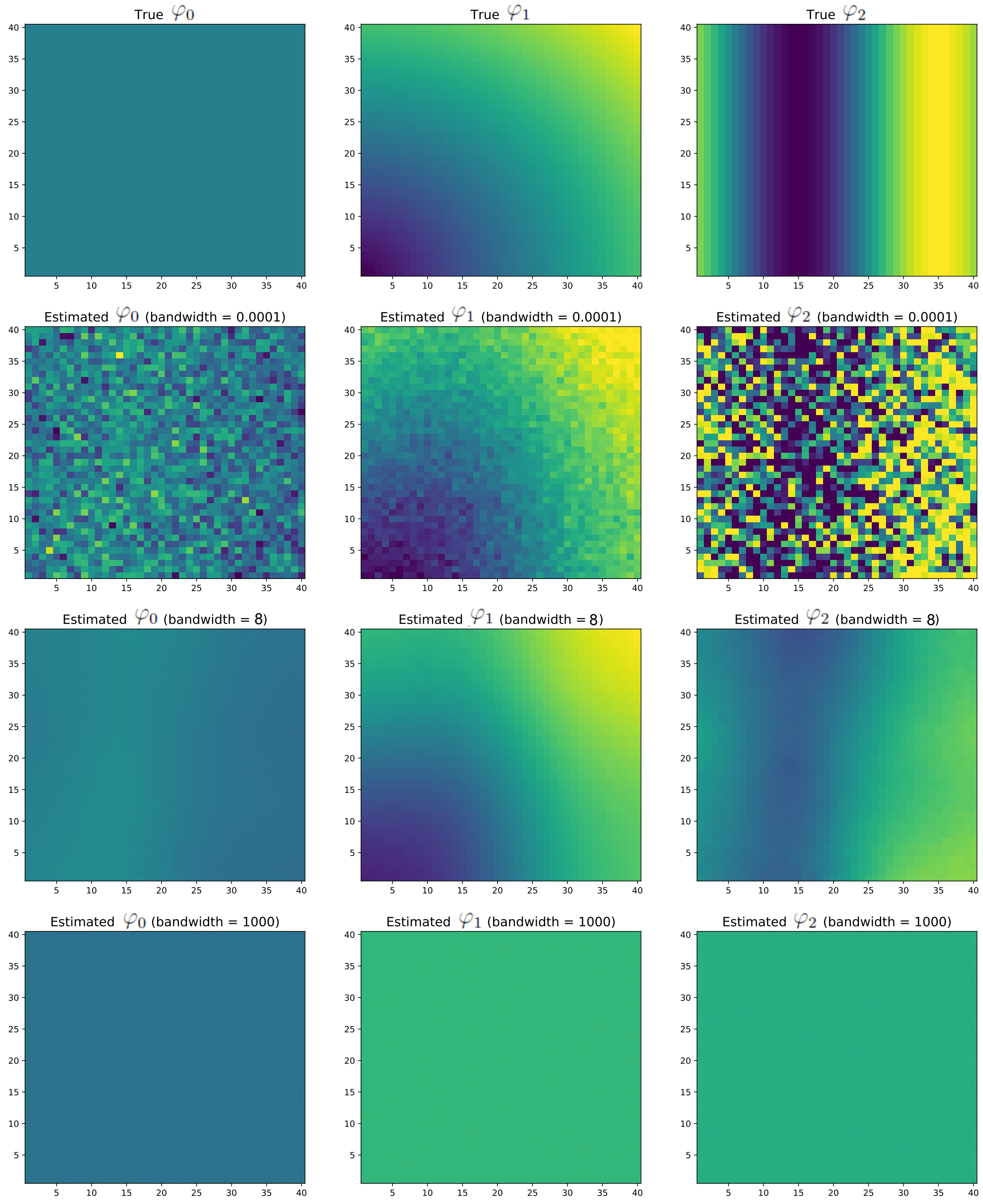

Figure A4: **Heatmap of the true values and estimated means for the coefficients φ_0, φ_1 and φ_2 by the Bayesian GWR model when number of observations is 50, with geographic bandwidth $\eta = 0.0001, 8$ and 1000.**

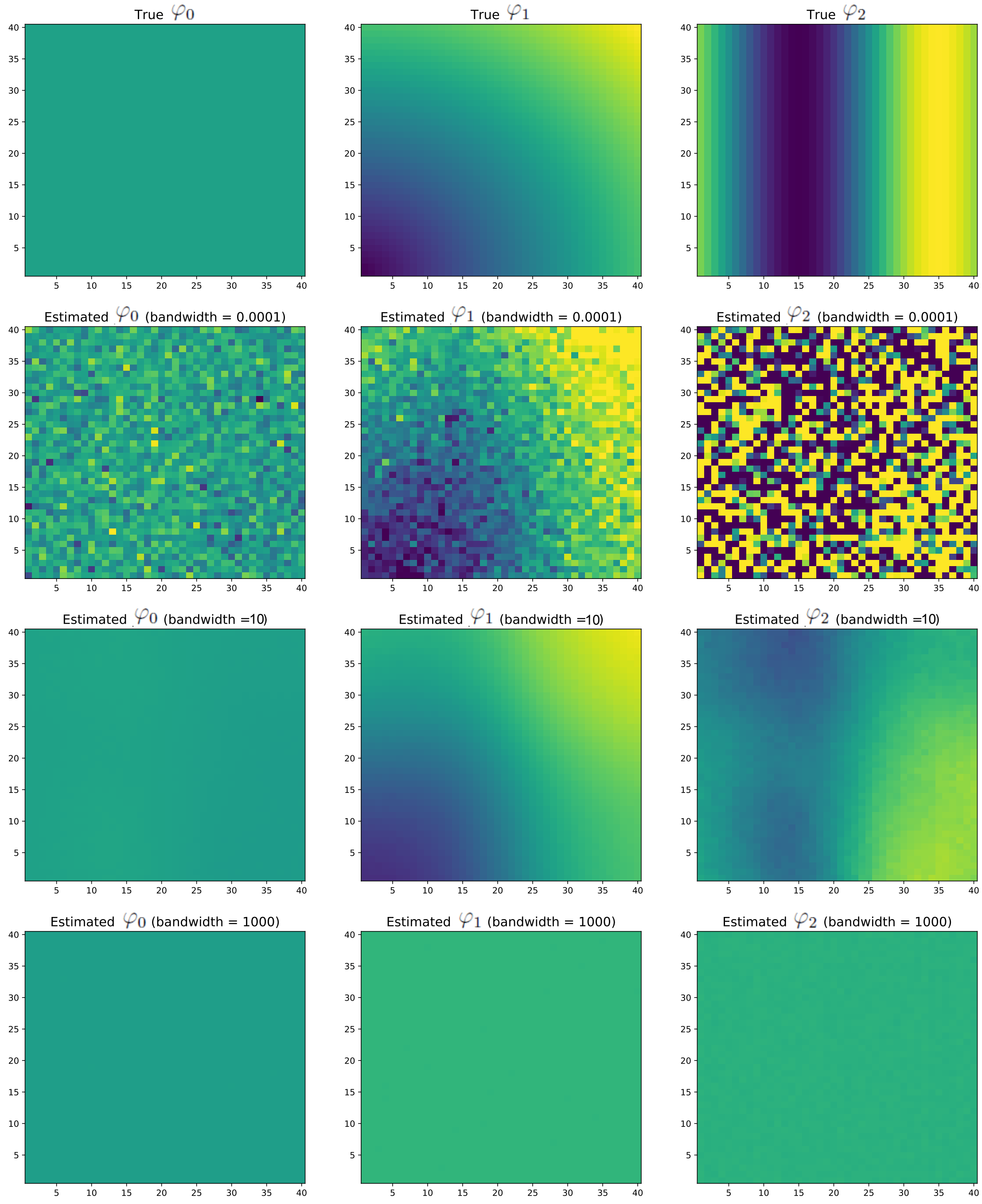

Figure A5: **Heatmap of the true values and estimated means for the coefficients φ_0, φ_1 and φ_2 by the Bayesian GWR model when number of observations is 10, with geographic bandwidth $\eta = 0.0001, 10$ and 1000.**

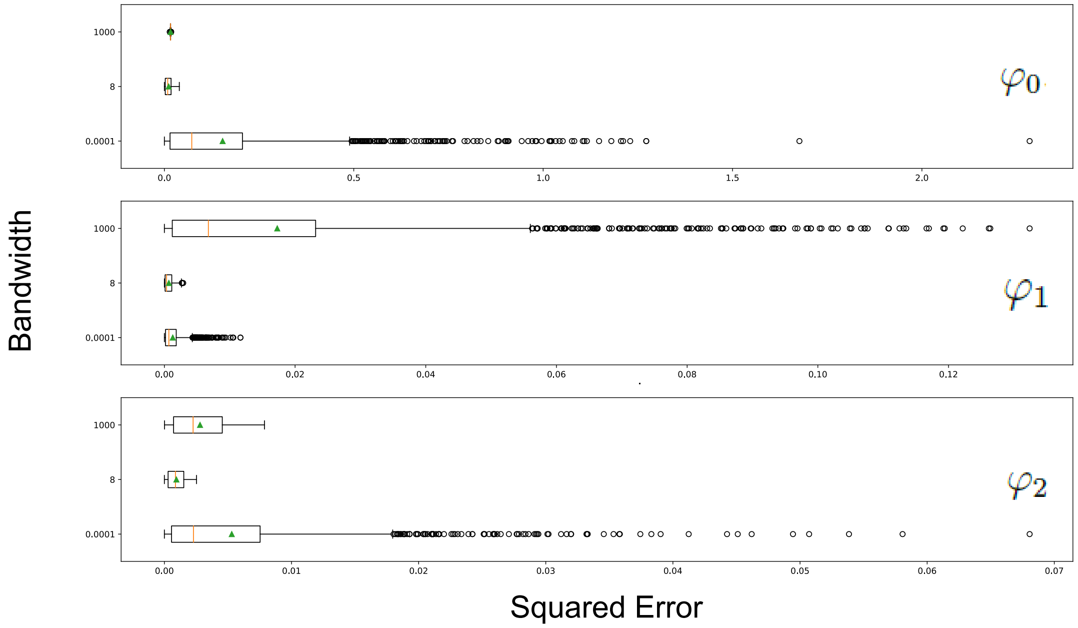

Figure A6: **Boxplots of the squared error of the estimated mean coefficients φ_0, φ_1 and φ_2 under the Bayesian GWR model across geographic locations, with geographic bandwidth $\eta = 0.0001, 8,$ and 1000, under 50 observations case.** The orange line and green triangle indicate the median and mean squared error.

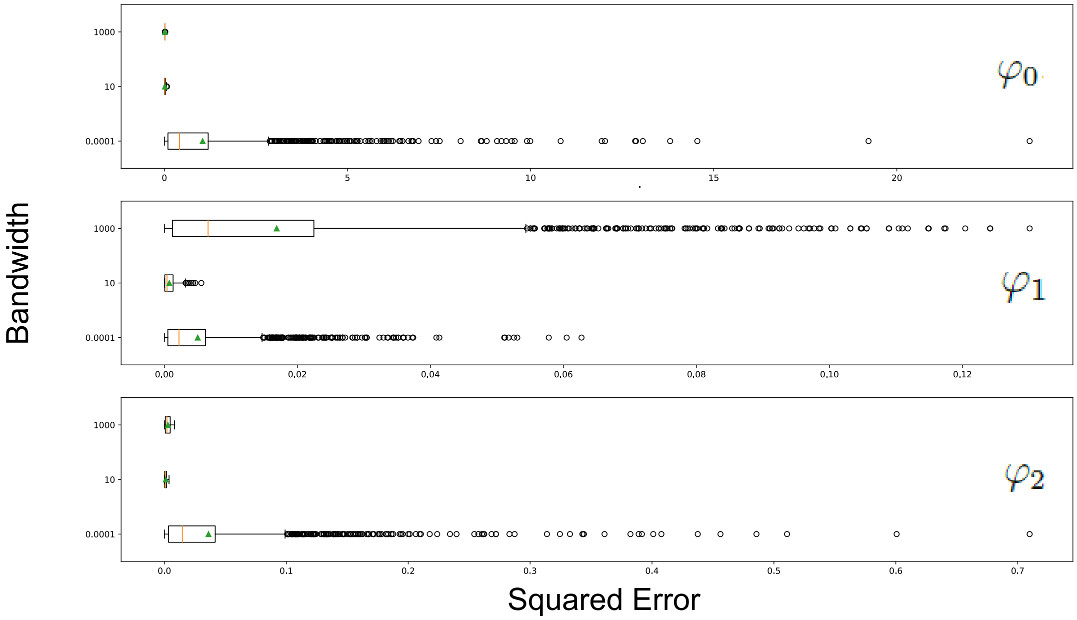

Figure A7: **Boxplots of the squared error of the estimated mean coefficients φ_0, φ_1 and φ_2 under the Bayesian GWR model across geographic locations, with geographic bandwidth $\eta = 0.0001, 10,$ and 1000, under 10 observations case.** The orange line and green triangle indicate the median and mean squared error.

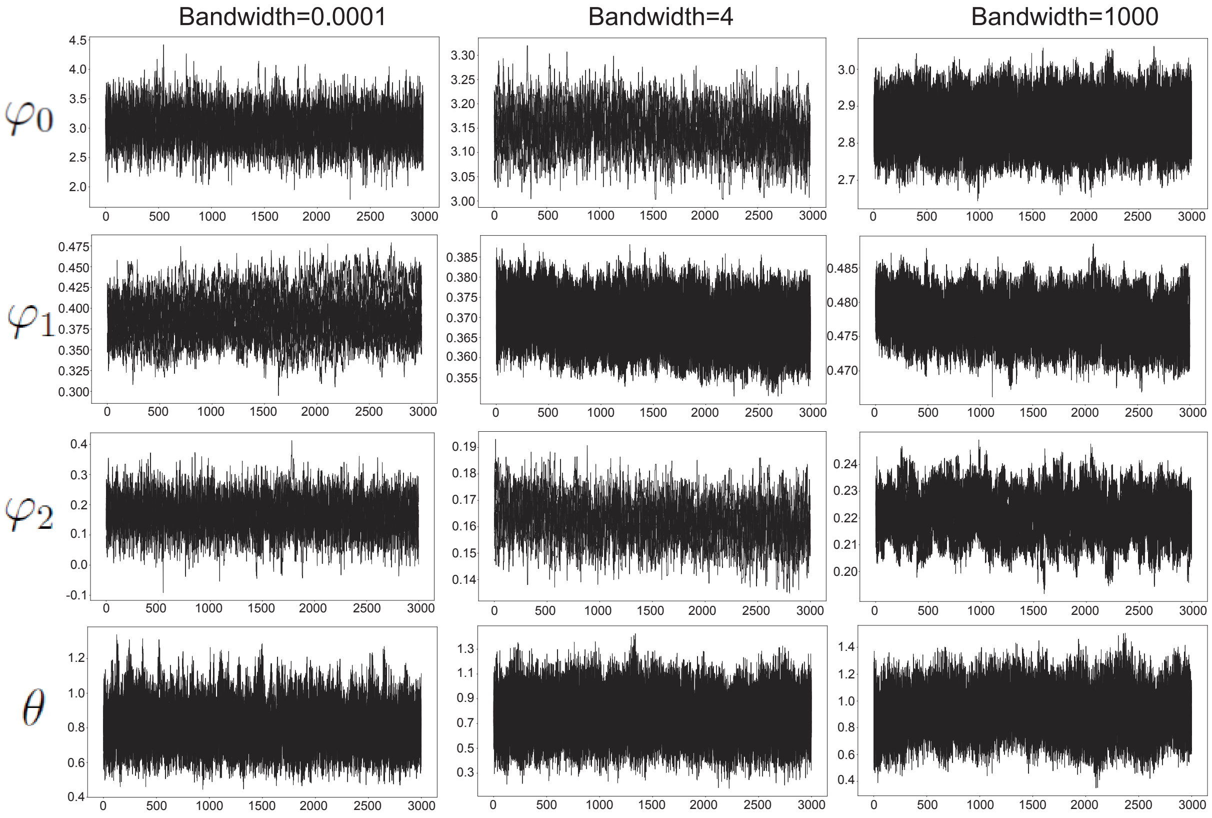

Figure A8: **Trace plot of SMI samples for φ_0, φ_1, φ_2 and θ when $\eta \in \{0.0001, 1, 20\}$.** Each plot contains results of 10 chains. The upper and lower bounds of trace plots reveal that the empirical Bayesian GWR posterior tends to have lower variance with higher geographical bandwidth.
